# Physical, chemical, and biological controls on algal dynamics in the Greenup Pool of the Ohio River

**DOI:** 10.1371/journal.pone.0360624

**Published:** 2026-10-26

**Authors:** Mindy Yeager-Armstead, Geneve Brady, Austin Hoffman

**Affiliations:** 1 Department of Natural Resources and Earth Science, Marshall University, Huntington, West Virginia, United States of America; 2 Environmental Health and Safety, Marshall University, Huntington, West Virginia, United States of America; NRSC: National Remote Sensing Centre, INDIA

## Abstract

Large river ecosystems are complex and difficult to study. Spatial and temporal variability in managed river ecosystems is further complicated by structural alterations such as channelization, dams, and hydroelectric power stations. The increasing frequency and intensity of harmful algal blooms in lotic systems challenges the convention that productivity in these systems is low. A substantial algal bloom in the Ohio River in the fall of 2015 with a lesser bloom in the fall of 2019 demonstrated the need for research in this area. To characterize algal dynamics in managed rivers, and specifically the Ohio River, high frequency sensors were deployed on lock structures of the Robert C. Byrd and Greenup Dams bracketing the 100-km Greenup pool and providing continuous monitoring of pool conditions. Depth profiles at transect sampling locations were conducted through the pool’s entirety to profile spatial variability. Parameters measured included light, turbidity, chlorophyll-a, phycocyanin, specific conductance, temperature, pH and dissolved oxygen. These data are combined with USGS discharge data and nutrient data from multiple sources to examine temporal and spatial conditions in the pool as they pertain to algal growth and productivity. High turbidity and subsequent light limitation constrain algal growth throughout most of the year. Lower flow conditions in the late summer months demonstrated higher light penetration and limited thermal stratification and were associated with higher algal biomass as conditions approach those of a lentic system. Chlorophyll-a, indicating green algae or diatoms, dominated the late summer and fall algal communities during this investigation. Cyanobacteria, indicated by phycocyanin measurements, showed peaks in winter months with cooler temperatures and reduced pool retention, possibly indicating tributary sources. These data will support the development of models to advance harmful algal bloom (HAB) predictions and evaluation of management options. Results may be relavent to other managed river systems.

## Introduction

Large river systems are understudied with respect to wadable streams and lake ecosystems primarily due to the complexity and difficulties in sampling. However, they are of immense importance to society due to our dependence on them for drinking water, fisheries, transportation, navigation, and recreation. While thought to lack substantial primary productivity due to current and turbidity [1,2], recent algal blooms in lotic ecosystems have raised questions as to conditions contributing to formation of the blooms. Concurrently, growing dead zones in coastal areas receiving large river discharge have prompted interest in decreasing nutrient loading and increasing nutrient uptake and retention in the riverine systems [3]. Despite efforts to control loading [4], nutrient rich runoff from agricultural and urban areas, as well as municipal wastewater inputs continue to impact large river systems. Riverine management is further complicated by many human disturbances associated with larger systems such as channelization and dredging, dams for navigation and hydroelectric stations [5], loss of floodplain connection, and watershed development. Reduced groundwater inflows and loss of riparian shading, coupled with rising global temperatures, further threaten these large systems where impacts are cumulative for the watersheds.

When flow volume on large rivers is altered by lock and dam structures to maintain navigational flow, control flooding, or support hydroelectric dams, riverine ecosystems are permanently altered. These managed or controlled rivers become more lentic with retention time in pools between locks and dams variable. Although more lentic, lake dynamics are not representative of controlled rivers; narrow channels, substantial flow volumes and velocities, turbidity, and frequent mixing by barges being unique to the controlled river systems. Consideration of this unique altered ecosystem is significantly relevant as most large rivers are now controlled for navigation and flood control [6].

Algal blooms are increasing globally as overallocated water resources are managed for competing uses, Phytoplankton blooms in lotic systems, are increasingly recognized and have been understudied with respect to lake and reservoir blooms. Large river ecosystems are known to have high sediment loads; turbidity and flow are expected to limit phytoplankton communities [7,8]. Despite the low probability, algal blooms are becoming more frequent and widespread in managed river ecosystems [5]. In the fall of 2015, an algal bloom developed over 650 miles of the Ohio River increasing drinking water treatment costs and impeding recreational activity over a two-month timespan. Levels of microcystin, a hepatotoxin produced by the dense growth of cyanobacteria algae, reached as high as 1,900 µg/L at river mile 468.8 near Cincinnati, OH [9] which is over 200 times higher than the USEPA’s recommended criteria for contact recreation [10]. More localized blooms have subsequently been reported [11,12]. As the frequency of harmful algal blooms (HABs) increases, the importance of understanding conditions in managed river systems, particularly those favoring algal growth and bloom formation, becomes critical. Each river, and each pool of a river, can be variable due to differences in retention times and velocities which vary based on the reservoir size and type (lake-like or run of the river) and the management strategy (i.e., hydroelectric, transportation, flood control) of the pool. These differences, in addition to seasonal patterns and watershed land use patterns, affect all aspects of the ecosystem including water velocity and turbidity, which may be significant factors in algal productivity and the potential for bloom development.

The objective of this research is to describe physical, chemical, and biological conditions in a managed river ecosystem and relate parameters to biological endpoints. With a focus on algal growth and HAB potential, we will characterize the variables potentially important in algal bloom development including the traditionally recognized variables, light, temperature and nutrients, and the additional variables in riverine systems, flow (velocity) and turbidity. Focusing on the Greenup Pool of the Ohio River, these factors will be characterized spatially by sampling transects at one meter depths and temporally using high frequency monitoring. Conditions will be related to algal community structure utilizing pigment concentrations as community proxies.

A description of the chemical and physical conditions in the Greenup Pool and the relationship between ecologically relevant parameters and algal productivity will build knowledge on the ecology of managed river systems. Outcomes mayprovide insight into factors contributing to algal growth and HAB development and potential management and mitigation strategies. Additionally, a nearly 4-year high frequency dataset is introduced which provides the foundation of this assessment and future endeavors.

## Materials & methods

### Site description

The Ohio River watershed extends through parts of Illinois, Indiana, Kentucky, Maryland, New York, North Carolina, Ohio, Pennsylvania, Tennessee, Virginia, and West Virginia (Fig 1). The total drainage area of the Ohio River Watershed is ~ 528,202 square kilometers with more than 25 million people, almost 10% of the US population, residing in the watershed. The Ohio River, itself, is an 8th order stream, flowing 1,579 kilometers from the confluence of the Allegheny and Monongahela Rivers in Pittsburgh, Pennsylvania and ending in Cairo, Illinois. Designated uses of the river include drinking water, recreation, aquatic life, warmwater fishery, and navigation. The river serves as drinking water for more than 5 million people. The Ohio River is also managed for flood control and navigation with 20 dams managed by the United States Army Corps of Engineers. The dams have reduced riverine velocity and created a series of pools with velocity and discharge mediated regionally by management decisions to maintain navigational pool levels and locally by operation of locking chamber dam structures. The magnitude of hydrologic restrictions are variable and range from little restriction during high flow to substantial restriction for maintenance of a navigational pool under low flow conditions. The Greenup Pool of the Ohio River spans 99.5 kilometers of the river from the Robert C. Byrd Locks and Dam near Apple Grove, WV, at river mile 279.2, to the Greenup Locks and Dam near Greenup, KY, at river mile 341.0. The downstream structure is a 70-megawatt run-of-river hydroelectric facility operated by the City of Hamilton, Ohio and American Municipal Power. Significant tributaries entering the river in the Greenup Pool include the Guyandotte, Mud and Big Sandy Rivers.

**Fig 1:**
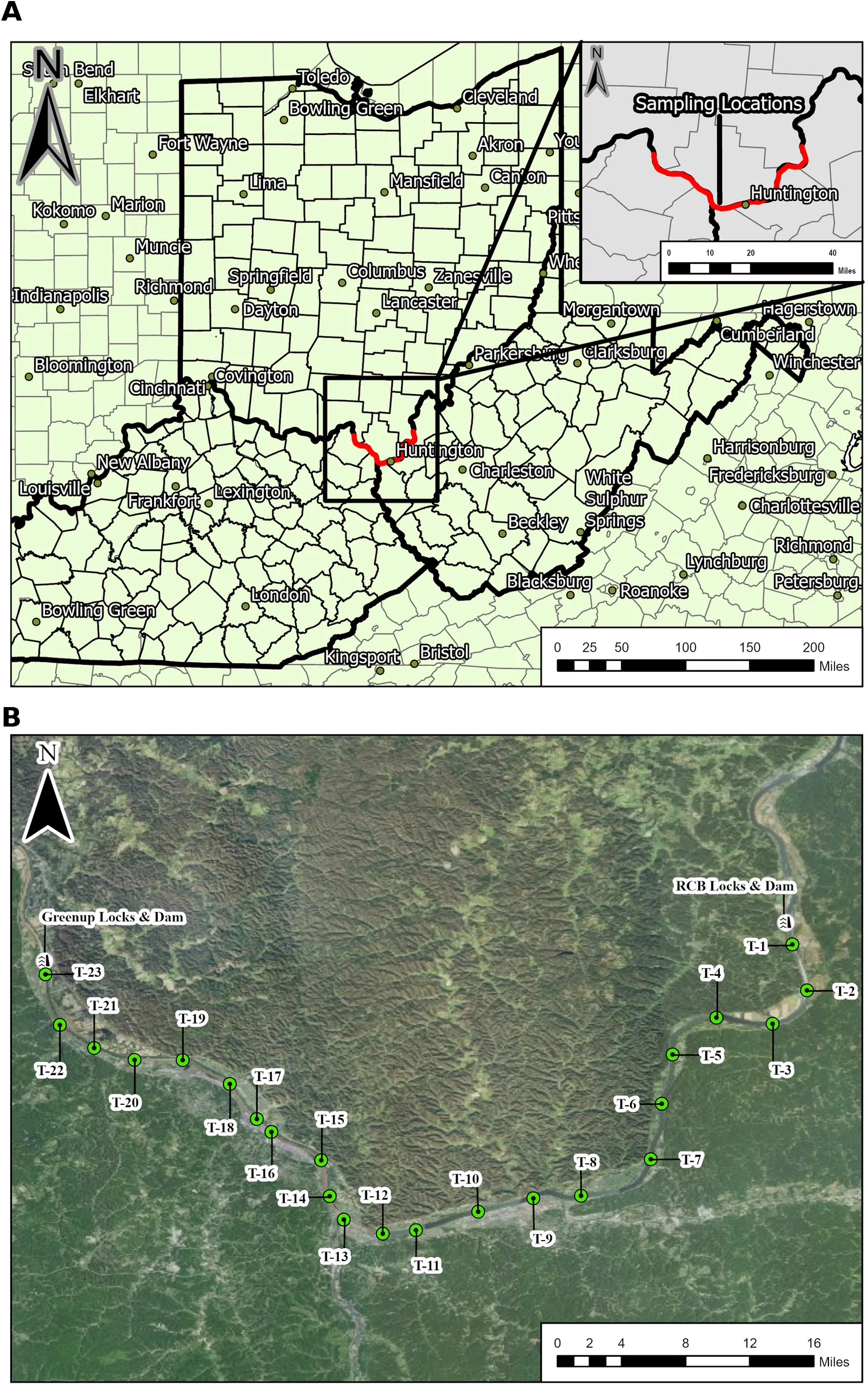
Study location and transect sampling locations in the Greenup Pool of the Ohio River. **(A)** General location of the Greenup Pool within the regional Ohio River watershed. **(B)** Transect sampling locations spanning the 99.5 km Greenup Pool from the R.C. Byrd Locks and Dam in Apple Grove, West Virginia, to the Greenup Locks and Dam in Greenup, Kentucky. Map layers were generated by the authors using public-domain geospatial data from the U.S. Geological Survey National Map imagery service, U.S. Geological Survey National Hydrography Dataset, and Natural Earth. Transect locations were generated from author-collected field coordinates.

### Data sources

Data used for the physical and chemical characterization of conditions in the Greenup pool were collected from multiple sources. Sensors were deployed and field sampling was conducted as described below. In addition, the United States Geological Survey operates a river gaging system for the Ohio River near Ironton, Ohio (03216070). The position of the gage is 38.5320556°, −82.6859444° and it is located at approximately river mile 327.1. This station records high frequency discharge from historical to present and was fitted with additional sensors in 2015 and 2020. Where indicated, these data were used for the current investigation. The Ohio River Valley Water Sanitation Commission (ORSANCO) collects bimonthly grab samples at both upstream and downstream lock and dam structures on the Greenup Pool of the Ohio River. These data are publicly available on the organization’s website and, where indicated, were utilized in the evaluations herein.

### Sensor deployment

To characterize water quality conditions a monitoring station for sensor deployment was established on the bulkhead of the Greenup Lock and Dam structure on the upstream side of the navigational structure. The bulkhead extends 480 meters upstream from the lock and is approximately 130 meters from the left bank. The channel is approximately 500 meters in width at that location. The deployment location was representative of the conditions in the main channel of the river, but it should be noted that the site best represents the lower end of the pool. As is described herein, the pool changes longitudinally from the shallower, more riverine upper end to the deeper, more lentic lower reaches. The meters were deployed in polyvinyl casing and rested approximately 1 meter below the water surface at base flow conditions. However, the meters were stationary, and water depth over the sensors would have been greater during high flow conditions. Water level, maintained for navigational flow, never fell below the sondes. Deployed instrumentation included a YSI EXO-2 sonde, a LI-COR photosynthetically active radiation (PAR) sensor, and a SUNA 2 nitrogen sensor. The sondes were set to record in 15-minute intervals. Dates of deployment and data quality objectives for each parameter monitored are shown in Table 1. Sensors were deployed and calibrated per the manufacturer’s instructions. Cleaning, calibration and data download generally occurred monthly by switching out the deployed instrument with a second calibrated sonde. A drift check was performed in the field between the newly calibrated instrument and the sensor being retrieved. The comparison was used to inform when probes needed replaced but data were not modified. The retrieved instrument was cleaned and recalibrated for return to the field. The nitrate sensor was prevented from biofouling with a wiper. The EXO-2 sensors were equipped with a wiper but it was not used due to the high turbidity in the river which lodged in the brushed and scratched the probes. Biofouling was minimized on the deployed EXO-2 sondes by the use of a copper sonde guard. In winter months, the deployment was longer due to high flow conditions limiting access to the bulkhead. The data set in its entirety is available through EPA’s Water Quality Exchange/Water Quality Portal under organization ID MARSHALL_UNIV_NRES. No permits were necessary for deploying the in-situ sensors, however access to the lock was granted by the US Army Corp of Engineers Huntington District, and the on-site lockmaster gave permission for accessing the site as requested.

**Table 1:** Parameters monitored at the Greenup Lock and Dam monitoring station and evaluated in transect sampling.

Sensor	Date(s)		Parameters	Units	DQO
	Deployed	Retrieved			
YSI EXO-2 Sonde	July 2017	December 2021	pH	PH mV	±0.1 pH units within ±10°C of calibration temp; ± 0.2 pH units for entire temp range
			Temperature	°C °F	−5–35°C: ± 0.01°C 35 to 50°C: ± 0.05°C
			Specific Conductivity	µS cm^-1^ mS cm^-1^	0-100 mScm^-1^: ± 0.5% of reading or 0.001 mScm^-1^, whichever is greater; 100−200 mScm^-1^: ± 1% of reading
			Turbidity	NTU	0-999 NTU: 0.3 NTU or ±2% of reading, whichever is greater; 1000–4000 NTU: ± 5% of reading
			Dissolved Oxygen	% Sat mg L^-1^	0-200%: ± 1% reading or 1% air sat., whichever is greater; 200-500%: ± 5% reading 0-20 mg L^-1^: ± 1% of reading or 0.1 mg L^-1^; 20−50 mg L^-1^: ± 5% reading
			Chlorophyll	RFU µg L^-1^	Chl-a: R2 > 0.999 for serial dilution of Rhodamine WT solution from 0–400 μg L^-1^ Chl-a equivalents
			Phycocyanin	RFU µg L^-1^	Phyco: R2 > 0.999 for serial dilution of Rhodamine WT solution from 0–100 μg L^-1^ phyco equivalents
SUNA V2	August 2019	December 2021	Nitratenitrite	mg L^-1^	±10% of reading or ±2 mg L^-1^-N, whichever is greater
LI-COR	August 2018; September 2018	December 2021	PAR	µmol s^1^ m^2^	7/3 microamps per 1000 µmol s^1^ m^2^

### Transect sampling

In addition to the high-frequency monitoring, a series of 22 transect locations representative of the pool including variable land uses, influence of major tributaries, and industrial outfalls were established (Fig 1b). It should be noted that 23 transect locations were originally proposed for sampling. One transect, T17, was not retained because the site did not provide additional information on variable land use, the influence of major tributaries, or industrial outfalls and site reductions were necessary. At each transect location, 5 locations across each transect were established equidistant across the river and sampled at multiple depths to monitor conditions in the water column at each location. One sampling event included only four transects due to sampling error. Under low flow conditions, transect sampling commenced in the summer of 2018 and continued in the summer of 2019. At each transect location, channel morphology and characteristics were recorded including width, depth, and bottom substrate composition. Secchi depth was recorded in triplicate and averaged. At each of the 5 (or 4) transect sampling locations depth was measured using a depth sensor. Samples and recorded measurements were taken at 0.5 m below the surface, 1 m intervals through the water column, and 0.5 m above the substrate. A YSI EXO-2 Sonde was used to record turbidity, pH, temperature, dissolved oxygen, chlorophyll-a (chlorophyll hereafter), and phycocyanin. Sensors data quality objectives are described in Table 1. A LICOR LI-192 underwater light sensor was affixed to the sonde, and PAR was similarly recorded. Water samples were collected from each depth using a Kemmerer bottle, and samples were split for additional water chemistry evaluation. To the extent practical, sampling was conducted at the transect locations over the 61.8-mile river segment under variable flow conditions, and visual surveys of bank conditions, inflows, and other observations were made to support pool characterization. No permits were necessary for the transect sampling as it was conducted by personal watercraft launched from publicly accessible boat ramps. No permits are required for the collection of water samples, algae or invertebrates.

For each vertical PAR profile, light attenuation with depth was calculated by I_z_ = I_0_e^-Kdz^, where I_z_ is PAR at depth z, I_0_ is PAR recorded 0.5 m below the surface, and K_d_ is the vertical light attenuation coefficient (m^-1^). For each transect-site profile, K_d_ was calculated as the negative slope of the linear regression between ln(PAR) and depth, where ln(PAR_z_)=ln(PAR_0_)-K_d_^Z^. Profiles with fewer than three valid PAR-depth measurements or non-positive PAR values were excluded from the attenuation calculation. The 1% light depth was calculated as z_1%_ = ln(100)/Kd. This value represents the depth at which PAR was reduced to 1% of estimated near-surface PAR and was used as an operational estimate of light availability in the water column. The calculated 1% light depth was paired with turbidity measured from the same transect-site profile to establish the relationship between turbidity and light in the river.

### Algal endpoints

The measured and calculated endpoints considered to represent the algal community and the influence of physical and chemical variables on community growth include chlorophyll (chl-a), phycocyanin (phyco), and the ratio of phyco to chl-a (BGGratio). The ratio of phycocyanin to chlorophyll was used as an indicator of changes in the algal community structure which may indicate increases in cyanobacteria and conditions favoring cyanobacteria over other taxa. Chlorophyll and phycocyanin were measured using the YSI EXO-2 Sonde and the bbe Algal Torch. Specifications for the EXO Sonde measurements are given in Table 1. The algal torch measures total chlorophyll and phycocyanin over a range of 0–500 µg chl-a L^-1^ with a 0.1 µg chl-a L^-1^ level of precision and 0–280 µg phyco L^-1^ with a similar level of precision. Measurements from the EXO Sonde and Algal Torch are used in the discussion to represent green algae (and diatoms) and cyanobacteria. Cyanobacteria did not reach nuisance levels, create visible algal blooms, or demonstrate sustained dominance during the data collection period to facilitate evaluation of environmental conditions supporting an algal bloom. However, the ratio of phyco to chl-a from the EXO Sonde or Algal Torch is used as a proxy for the ratio of cyanobacteria to green algae as an indication of conditions favoring increases in cyanobacteria over green algae or diatom growth. Specifically, a higher cyanobacteria:green algae ratio may indicate cyanobacteria are rising in relation to the green algae and diatoms measured as chl-a. It should be noted that pigment adaptation may contribute to this apparent community shift. Further investigation of trends is warranted.

### Data analysis

Forty-four months of data are summarized seasonally from the Greenup Pool of the Ohio River. Daily data recorded in 15-minute increments were summarized to daylight averages utilizing the 05:00–17:00 values. Analysis of mean, median, maximum and single time points indicated no apparent trends except the weak diurnal dissolved oxygen curve. Since daily maximums did not correspond to a single time point with mixing seeming to play a large role in homogeneity, averages of daylight hours were selected as representative of pigment concentrations and predictor variables.

When averages are reported, pH was converted to the hydrogen ion concentration for average calculation. However, pH values were utilized in the statistical analysis due to the hydrogen ion concentrations being below the threshold of values recognized by the statistical software. Missing data were ignored in correlation and Freidman analysis resulting in unequal but greater sample sizes. The multiple regression model required that no blanks be included in the dataset so lines with blanks were removed. Nitrate measurements from the SUNA had a limited temporal dataset compared the EXO sonde data and resulted in fewer data points in the regression analysis. However, due to the importance of nutrients to the algal endpoints and confidence in the 88–135 data point sample size, nitrate was retained in the analysis. Pool retention times were calculated by dividing pool volume by daily discharge to produce retention time in days. Pool volumes were measured during transect sampling and more accurately reflect the low flow conditions under which sampling was conducted than the higher flow conditions. Daily discharge rates were reported by the USGS gage at Ironton.

Seasonal multiple regressions were used to determine the significance of environmental drivers on the biological endpoints. Seasons were defined astronomically by solstices and equinoxes. Data were screened for collinearity and variance inflation factors (VIFs) were generally in the acceptable range (S1 Table). Although several VIFs were greater than 5, they were well below 10, the level indicating severe collinearity [13] and were associated with variables important in the analysis, so they were retained. Further, correlation coefficients of the predictor variables were all below the threshold value (|r| ≥ 0.70) of when collinearity would be expected to affect the model [14] (S2 Table). Autocorrelation was found to be significant in the dataset as indicated by autocorrelation function plots, Durbin Watson statistics and Lag residual correlations (S3 Table). Use of an auto regressive model for multiple regressions provided sufficient correction to interpret the analysis. Heteroscedasticity was demonstrated in seasonal analysis using Breusch-Pagan test and it was not substantially improved by dependent variable transformation (S4 Table) therefore the multiple regression with serial correlation proceeded without data transformation. General least squares techniques such as the multiple regression are an appropriate alternative when heteroscedasticity is present but other assumptions are met [15] as was the case when the autoregressive correction was added to the model.

For significance testing, data were checked for normality and homogeneity and generally did not meet those criteria. Spearman correlations and Friedman two-way analysis of variance by ranks with least significant difference multiple comparisons were used to determine correlations and significant differences between the groups.

Data diagnostics including VIF, correlation coefficients and auto correlations were performed in Lumivero Neural tools software (Version 8.12.0). Breusch-pagan testing was performed using publicly available python code from Statsmodels [16]. All additional analysis including multiple regressions with Cochrane-Orcutt serial correlation adjustments, Durbin-Watson tests, Spearman Rank Correlations, Friedman’s rank tests and the associated normality and homogeneity testing were performed in NCSS Statistical Analysis and Graphics software version 21.0.8.

## Results

### Physical and chemical conditions

The Greenup Pool of the Ohio River is 99.5 km long, ranged from about 375–510 meters in width, and 4–8 meters in depth from the upstream to downstream extent in the transect sampling conducted during the summers of 2018 and 2019 (Table 2). Daily flow averages in the Greenup Pool during the sampling period (8/27/2017 to 04/09/2021) ranged from 6,970–492,000 CFS with an average of 87,969 CFS (197–13,932 m³ s^−1^, with an average of 2,491 m³ s^−1^). Flow exhibited seasonal variability with the highest flows in spring (March and April), and the lowest flows in August, September, and October (Fig 2a). Retention times in the pool, estimated using pool volume and discharge, ranged from less than a day to just over 23 days. An average retention time was estimated to be 3.5 days with retention time less than 1 day approximately 20% of the time (Fig 2b). Pool retention was estimated to be greater than 3 days 60% of the time and greater than 10 days 5% of the time. There was significantly longer retention time during summer as compared to spring and winter and fall as compared to winter (Table 3).

**Table 2:** Physical and chemical conditions at transect locations in the Greenup Pool of the Ohio River on multiple sampling dates.

Transect 2				
Date	8/29/2018	7/2/2019	8/6/2019	9/16/2019
Width (m)	380	385	400	375
Discharge (CFS)	24,600	95,000	16,500	33,600
Avg Secchi Depth (m)	0.714	0.423	1.318	0.858
Algal Torch Chl-a (ug L^-1^)	3.814	2.691	4.557	2.019
Algal Torch Cyano (ug L^-1^)	0.201	0.115	0.233	0.126
TSS (mgL^-1^)	8.790	11.096	3.550	--
**Transect 7**				
Date	7/2/2018	7/2/2019	8/6/2019	9/16/2019
Width (m)	404	408	396	392
Discharge (CFS)	55,600	95,000	16,500	33,600
Avg Secchi Depth (m)	0.165	0.378	1.698	1.518
Algal Torch Chl-a (ug L^-1^)	2.096	2.676	4.624	1.465
Algal Torch Cyano (ug L^-1^)	0.192	0.102	0.294	−0.032
TSS (mg L^-1^)	59.000	23.248	2.508	--
**Transect 13**				
Date	7/11/2018	7/10/2019	8/20/2019	9/18/2019
Width (m)	500	490	495	496
Discharge (CFS)	29,300	86,700	20,800	25,900
Avg Secchi Depth (m)	0.599	0.148	1.218	1.506
Algal Torch Chl-a (ug L^-1^)	6.396	2.535	5.811	1.639
Algal Torch Cyano (ug L^-1^)	0.175	0.227	0.443	0.106
TSS (mg L^-1^)	11.145	104.465	--	--
**Transect 19**				
Date	8/15/2018	7/10/2019	8/20/2019	9/18/2019
Width (m)	410	388	384	379
Discharge (CFS)	37,300	86,700	20,800	25,900
Avg Secchi Depth (m)	0.8285	0.332	0.95	1.018
Algal Torch Chl-a (ug L^-1^)	9.551	2.423	5.480	2.196
Algal Torch Cyano (ug L^-1^)	0.176	0.132	0.401	0.110
TSS (mg L^-1^)	9.790	16.713	--	--
**Transect 23**				
Date	7/23/2018	7/10/2019	8/20/2019	9/18/2019
Width (m)	500	n/a	510	500
Discharge (CFS)	31,200	86,700	20,800	25,900
Avg Secchi Depth (m)	0.865	0.76	0.716	0.87
Algal Torch Chl-a (ug L^-1^)	2.726	2.385	5.617	1.588
Algal Torch Cyano (ug L^-1^)	0.147	0.064	0.583	0.069
TSS (mg L^-1^)	8.570	35.824	--	--

**Table 3:** Significant seasonal differences from Friedman two-way ANOVA by year and season for the 3 years with complete data sets. The non-parametric procedure found no significant differences between 2018, 2019, and 2020 for the variables tested.

	Q	p	Season	n	mean	Significant differences
Retention	8.2	0.04	Fall	227	−1.45	Winter
			Spring	277	−8.61	Summer
			Summer	273	3.26	Spring, Winter
			Winter	203	−12.89	Fall, Summer
Temp	9.0	0.03	Fall	266	14.20	Spring, Summer, Winter
			Spring	277	15.86	Fall, Summer, Winter
			Summer	273	26.97	Fall, Spring, Winter
			Winter	214	5.078	Fall, Spring, Summer
Turbidity	9.0	0.03	Fall	266	23.21	Spring, Winter
			Spring	277	38.36	Fall, Summer
			Summer	273	20.24	Spring, Winter
			Winter	214	52.80	Fall, Summer
Conductivity	4.2	0.240	Fall	266	336.77	--
			Spring	277	280.62	--
			Summer	273	358.86	--
			Winter	214	272.48	--
DO Saturation	9.0	0.03	Fall	266	91.15	Spring, Summer, Winter
			Spring	277	95.43	Fall, Summer
			Summer	273	86.04	Fall, Spring, Winter
			Winter	214	98.28	Fall, Summer
Green algae	7.0	0.072	Fall	266	1.84	--
			Spring	277	2.88	--
			Summer	273	2.57	--
			Winter	214	1.93	--
Cyanobacteria	6.6	0.086	Fall	266	0.36	--
			Spring	277	0.51	--
			Summer	273	0.10	--
			Winter	214	0.72	--

**Fig 2:**
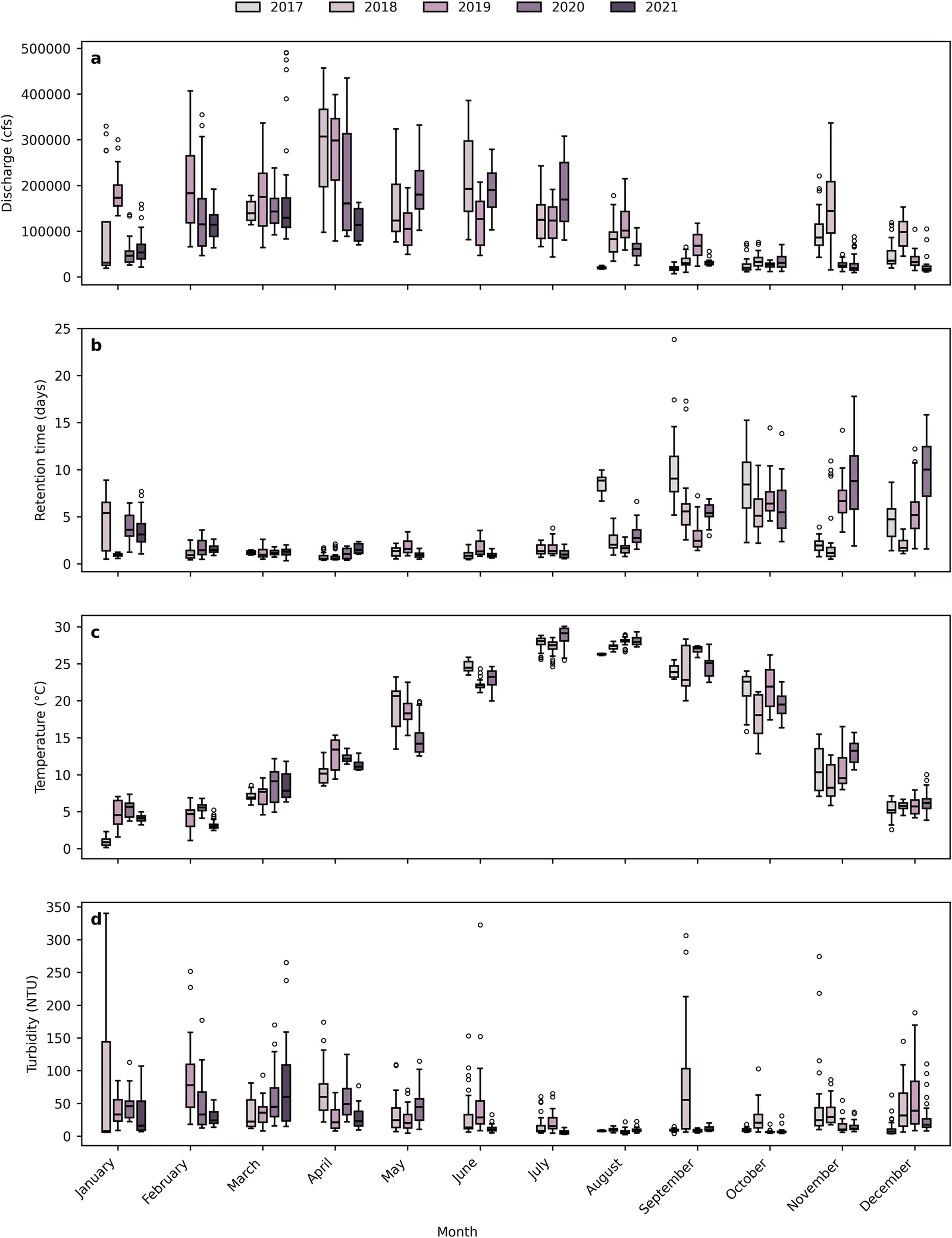
Physical conditions recorded by high frequency monitoring at the Greenup Locks (temperature, turbidity) and the USGS gaging station at Ironton (flow). Pool retention was calculated using the pool volume and discharge.

Temperature showed strong seasonal trends with the highest temperatures in July and August, 27.9°C and 27.7°C, respectively (Fig 2c). January and February had the lowest temperatures, averaging 4.2°C and 4.4°C, respectively. Annual temperatures range from 0°C to 30°C with an annual average of 15.3°C. Despite the similar ranges between spring and fall, all 4 seasons were significantly different than each other (Q = 9.0, p = 0.03; Table 3).

Annual average turbidity was 32.3 NTU with a range of 2.56 to 340.0 NTU. Although high turbidity occurred in all seasons, lowest turbidity values occurred in August, September and October, corresponding to the lowest flow conditions (Fig 2d). Turbidity was significantly lower in the summer and fall, as compared to spring and winter (Table 3). Turbidity was > 10 NTU 70% of the time and >5 NTU 95% of the time. Specific discussion of turbidity in relation to chemical and biological parameters is included below.

Transect sampling at 22 locations in the Greenup Pool in lower flow summer periods of 2018 and 2019 demonstrated spatial variability in the pool. Depth increased in a downstream direction from T1 to T23 (Fig 3) going from approximately 4–8 meters. River width increased from 345 to 500 meters over 99.5 kilometers. The water column distribution of temperature (Fig 3) and turbidity (Fig 4) indicate fairly uniform mixing, but stratification was noted primarily in the lower reaches under low flow conditions. Stratification is defined as a temperature differential greater than 0.5^o^C [17]. On August 6, 2019, thermal stratification was demonstrated at T-2, an upstream sampling location. In general, differences between surface and benthic temperatures were more frequent at the deeper downstream locations above the lock structure. Turbidity followed a similar trend to temperature with fairly uniform mixing throughout most of the year. Lower turbidity readings occurred under low flow conditions. Transect sampling indicated stratification of the water column sediment load in the summer of 2019 at the deeper, downstream end of the Greenup Pool.

**Fig 3:**
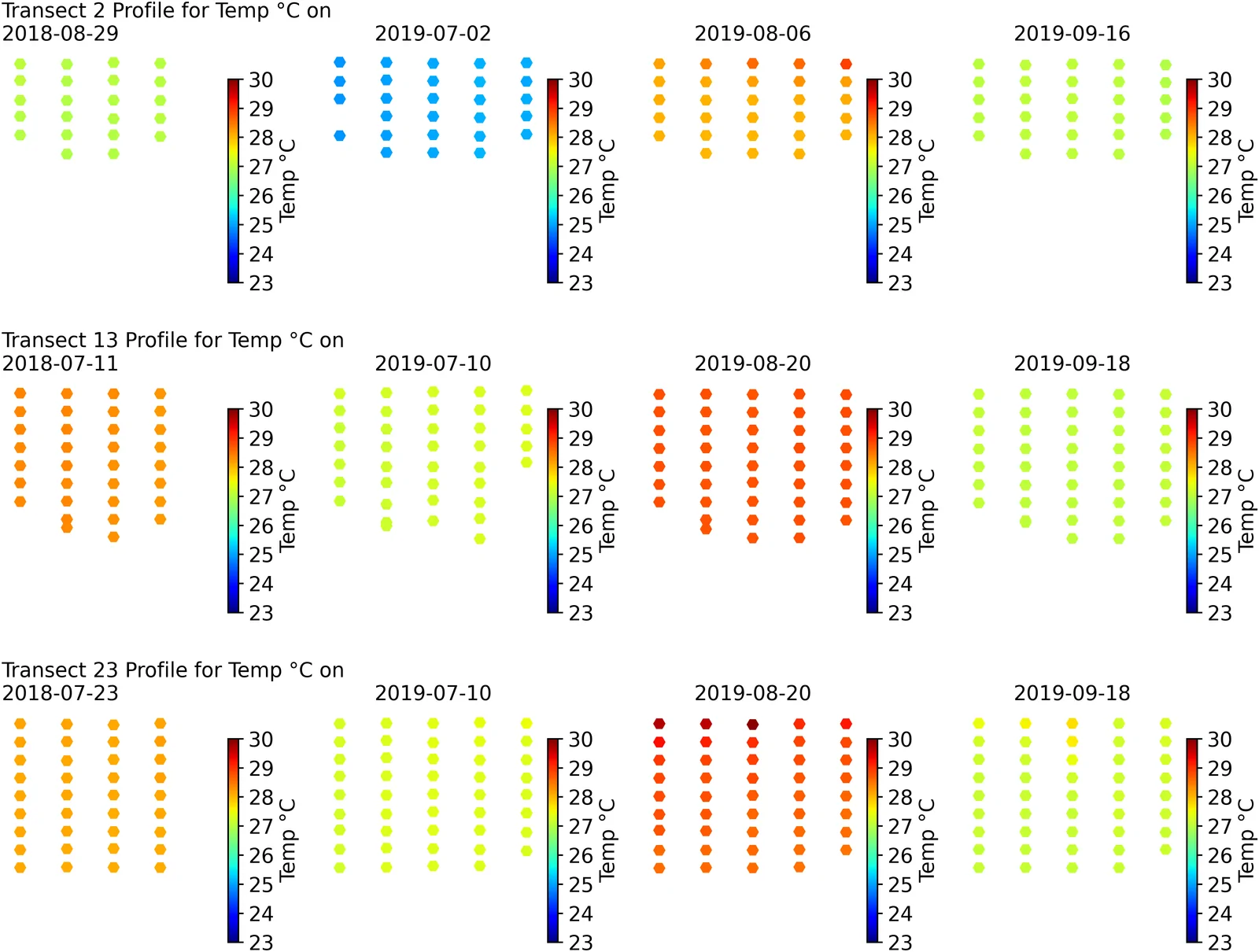
Spatial and temporal variability in temperature from transect sampling in the Ohio River under low flow conditions in the summer of 2018 and 2019.

**Fig 4:**
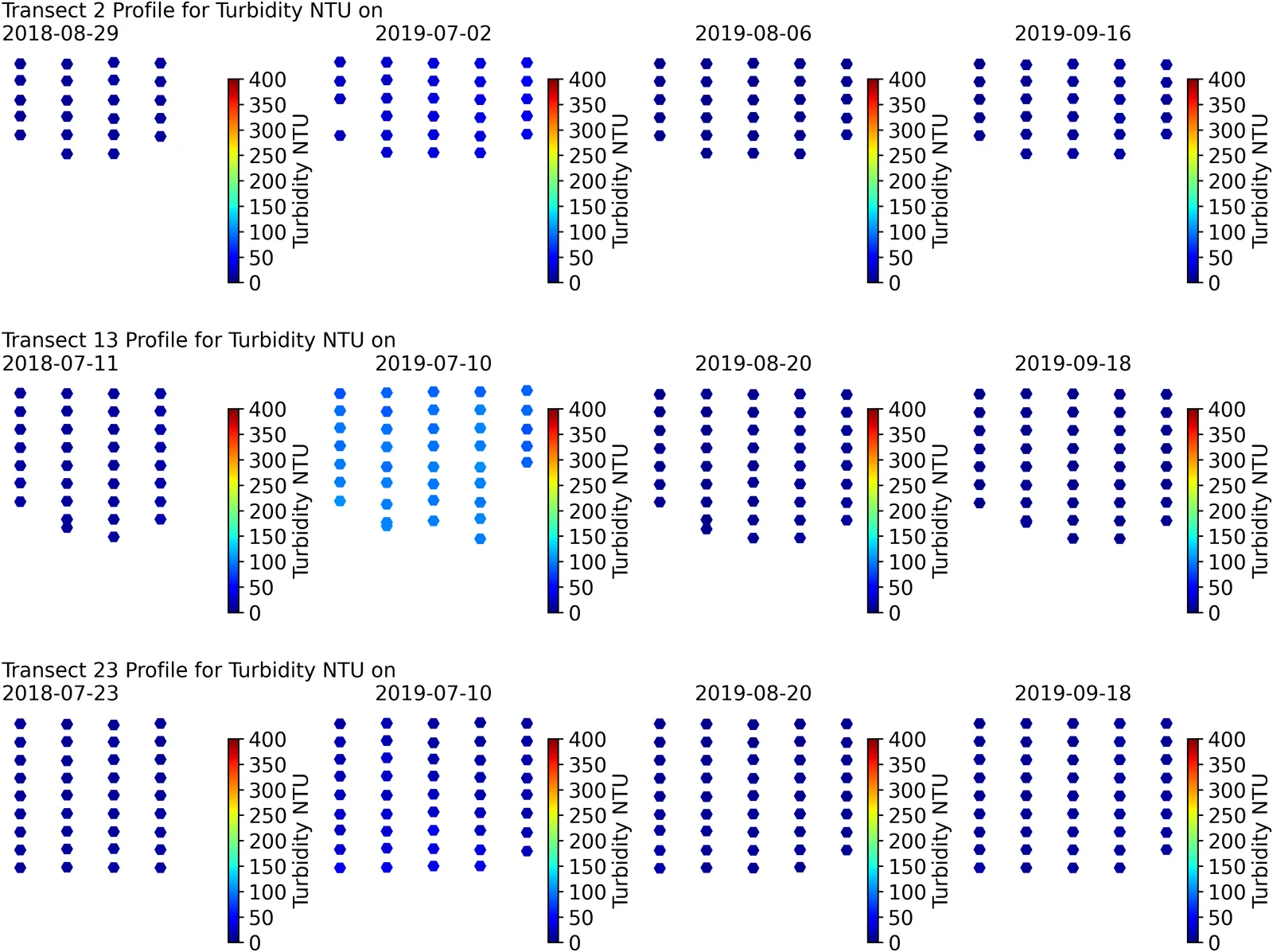
Spatial and temporal variability in turbidity from transect sampling in the Ohio River under low flow conditions in the summer of 2018 and 2019.

Even in low flow summer months, light was quickly attenuated in the water column (Fig 5) with Secchi depth averaging 0.47 meters and ranging from 0.1 to 1.70 meters (Table 2). The light attenuation coefficients (K_d_) ranged from 0.655 to 6.250 m^−1^, with a mean of 1.829 m^−1^ across retained PAR-depth profiles (R² ≥ 0.90; n = 184). The 1% light depths ranged from 0.737 to 7.030 m, with a mean of 3.188 m and showed a strong inverse relationship with turbidity (Fig 6).

**Fig 5:**
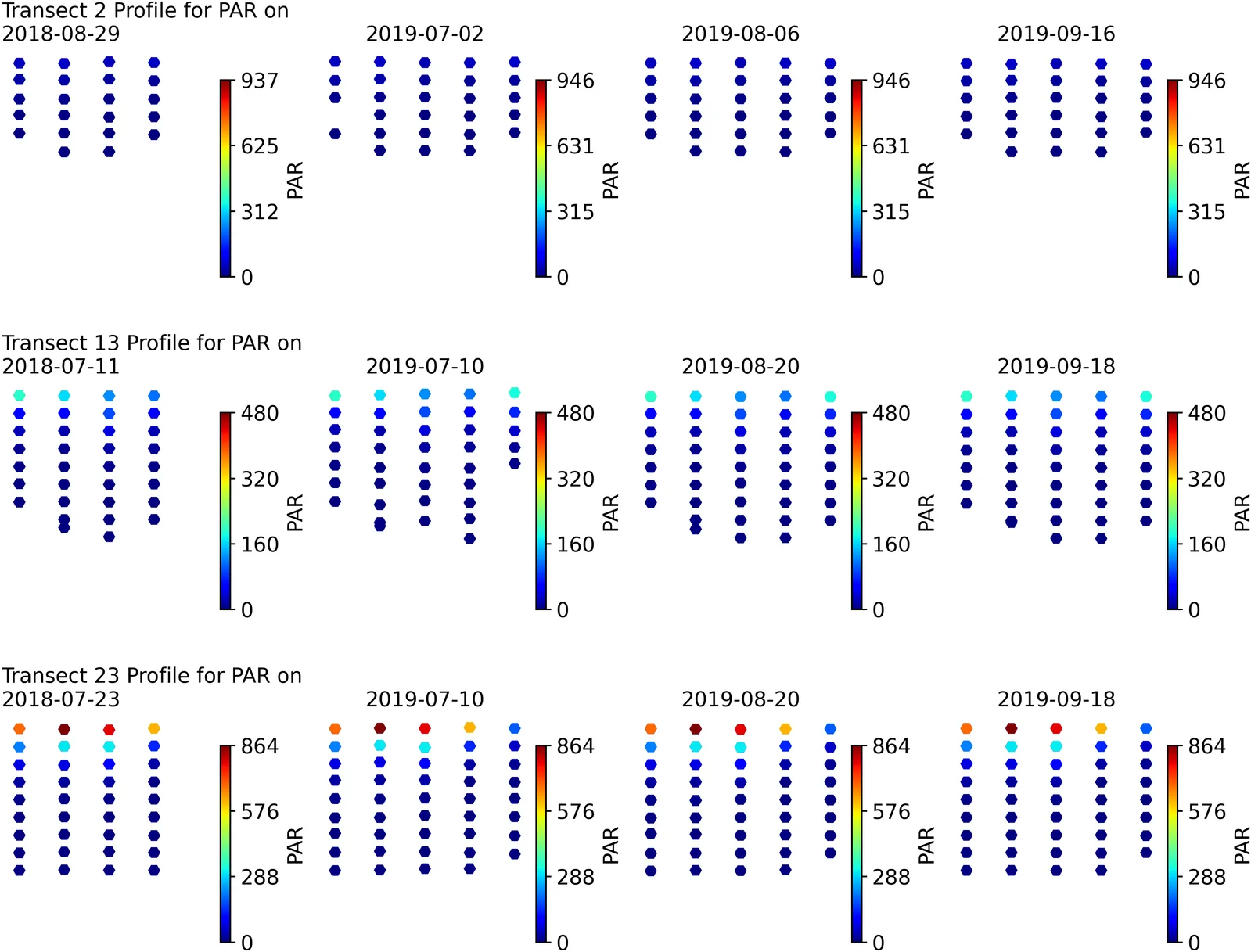
Differences in depth and velocity in the pool are demonstrated by PAR differences with increasing light penetrations in a downstream direction.

**Fig 6:**
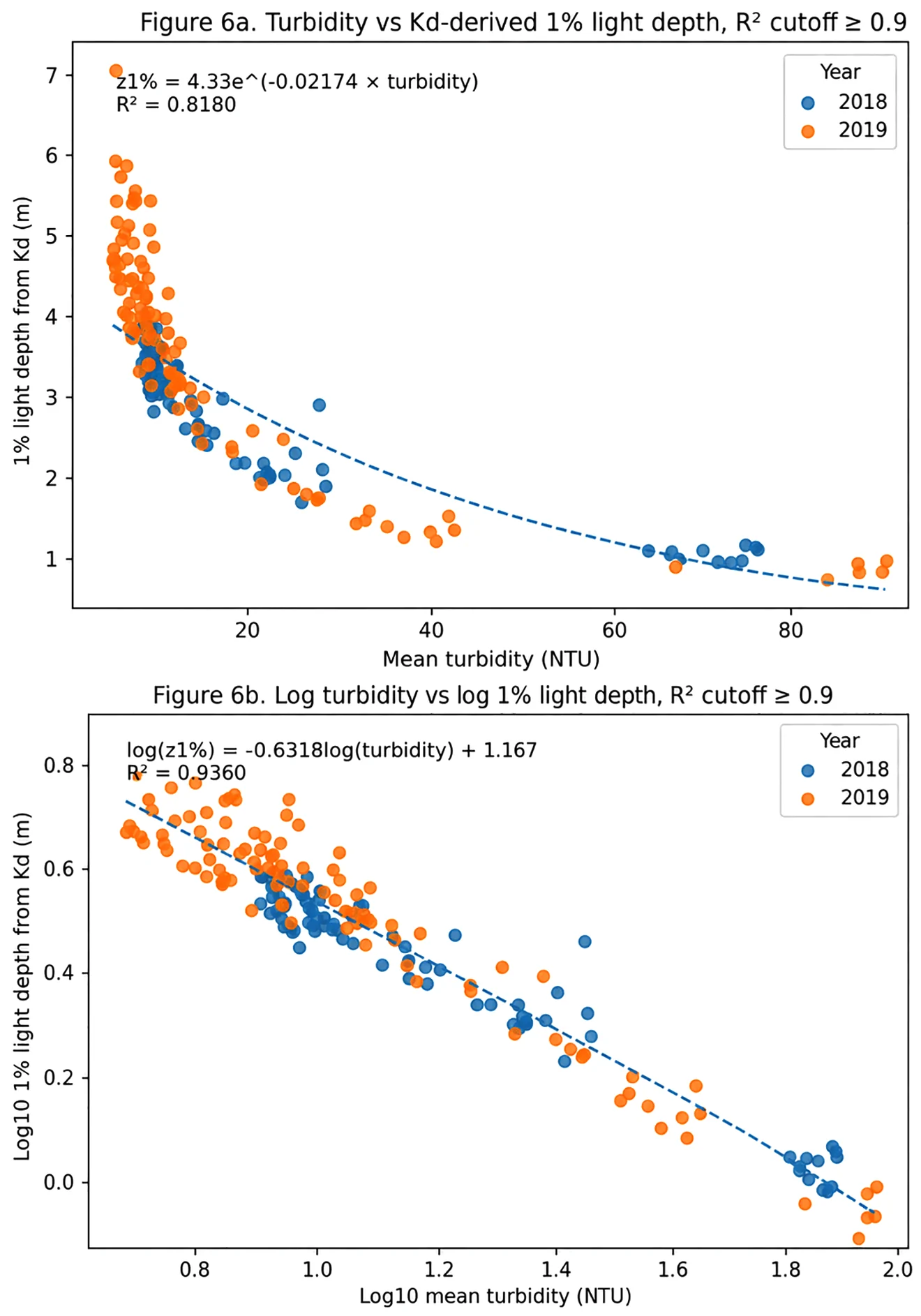
Relationship between light and turbidity from sampling stations in the Greenup Pool of the Ohio River from sampling dates in the summers of 2018 and 2019. Fig 6a shows the light depth and turbidity relationship with 6b showing the log relationship.

Water chemistry monitoring included dissolved oxygen concentration and percent saturation, specific conductivity, and pH. Dissolved oxygen concentrations exhibited strong seasonal patterns (Fig 7a) and were significantly lower in the summer than the other seasons (Table 3). The minimum values recorded were in September 2018 with 3 days of values measuring 5.2 to 5.3 mg L^-1^. The maximum value recorded was 14.5 mg L^-1^ with an average of 9.2 mg L^-1^ over the study period. Seasonal trends for the saturation of oxygen were less well defined (Fig 7b). Oxygen saturation ranged from 61.4 to 133%, averaging 93.2% overall. The highest saturation values occurred in July and August (Fig 7b); lowest saturation values occurred in August and September which were the highest temperature, and lowest flow months. Conductivity also peaked in September and October (Fig 7d), with the maximum value recorded on October 25, 2017. Conductivity ranged from 106.3 to 511.7 uS cm^-1^ with an average of 317.2 uS cm^-1^. Seasonal differences were noted but were not significant (Table 3). The pH was fairly consistent, averaging 7.68 SU and ranging from 7.28 to 7.92 SU 95% of the time. However, pH was elevated, between 7.92 and 9.3 SU for 5% of the time in the daily data with the elevations corresponding to summer and fall conditions.

**Fig 7:**
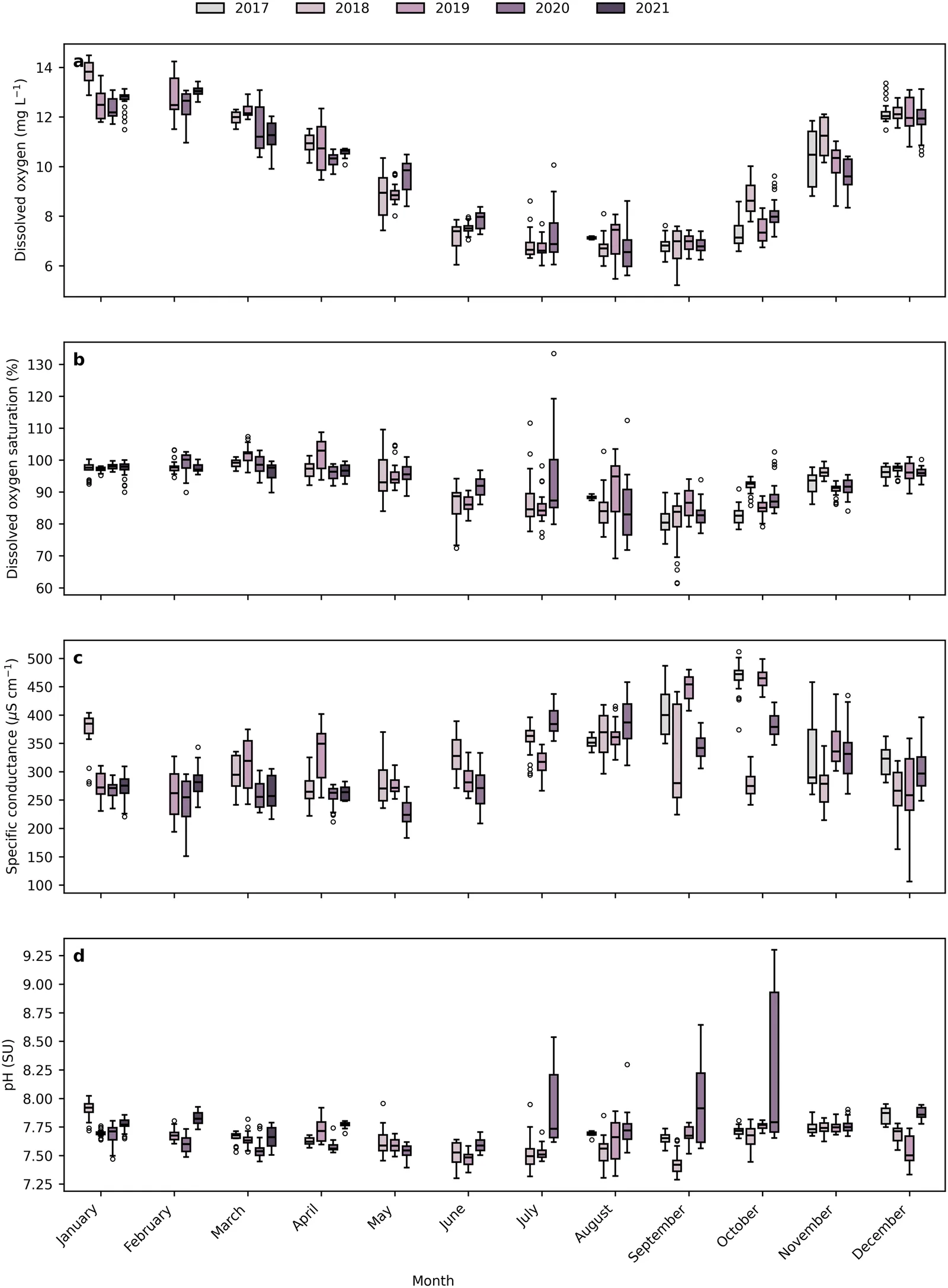
Seasonal variability of water chemistry parameters recorded by high frequency monitoring at Greenup Locks over 44 months. Graph depicts dissolved oxygen in mg L^-1^ (a) and % saturation (b), specific conductance (c) and pH (d).

The average nitrate was 0.71 mg L^-1^, and ranged from 0.41 to 0.94 mg L^-1^ over the sampling period with the lowest values recorded in May of 2020 (Fig 8). The deployed sensor measured nitrate-nitrite, but data are presented here as nitrate since that is the dominant form. ORSANCO bimonthly nitrogen, ammonia, and phosphate grab samples from January 2017 to March of 2023 are mentioned here to give a complete view of conditions in the pool. Nitrate-nitrogen averaged 0.83 mg L^-1^ and ranged from 0.92 mg L^-1^ to 2.94 mg L^-1^ in the 17-year dataset of bi-monthly grab samples. Ammonia-nitrogen averaged 0.058 mg L^-1^ and ranged from 0.020 mg L^-1^ to a high of 0.057 mg L^-1^ with the highest values as outliers and 90% of the values less than 0.100 mg L^-1^. Phosphate averaged 0.086 mg L^-1^ and ranged from 0.010 to 0.640 mg L^-1^ with non-detects noted in each of the 4 seasons. Similar to nitrate trends from the current study, ORSANCO nutrient data indicated nitrate, ammonia and phosphate were generally highest in the winter months and lowest in the summer and fall. The ORSANCO data were not combined with our data for analysis but are included to provide additional data for interpretation of riverine conditions.

**Fig 8:**
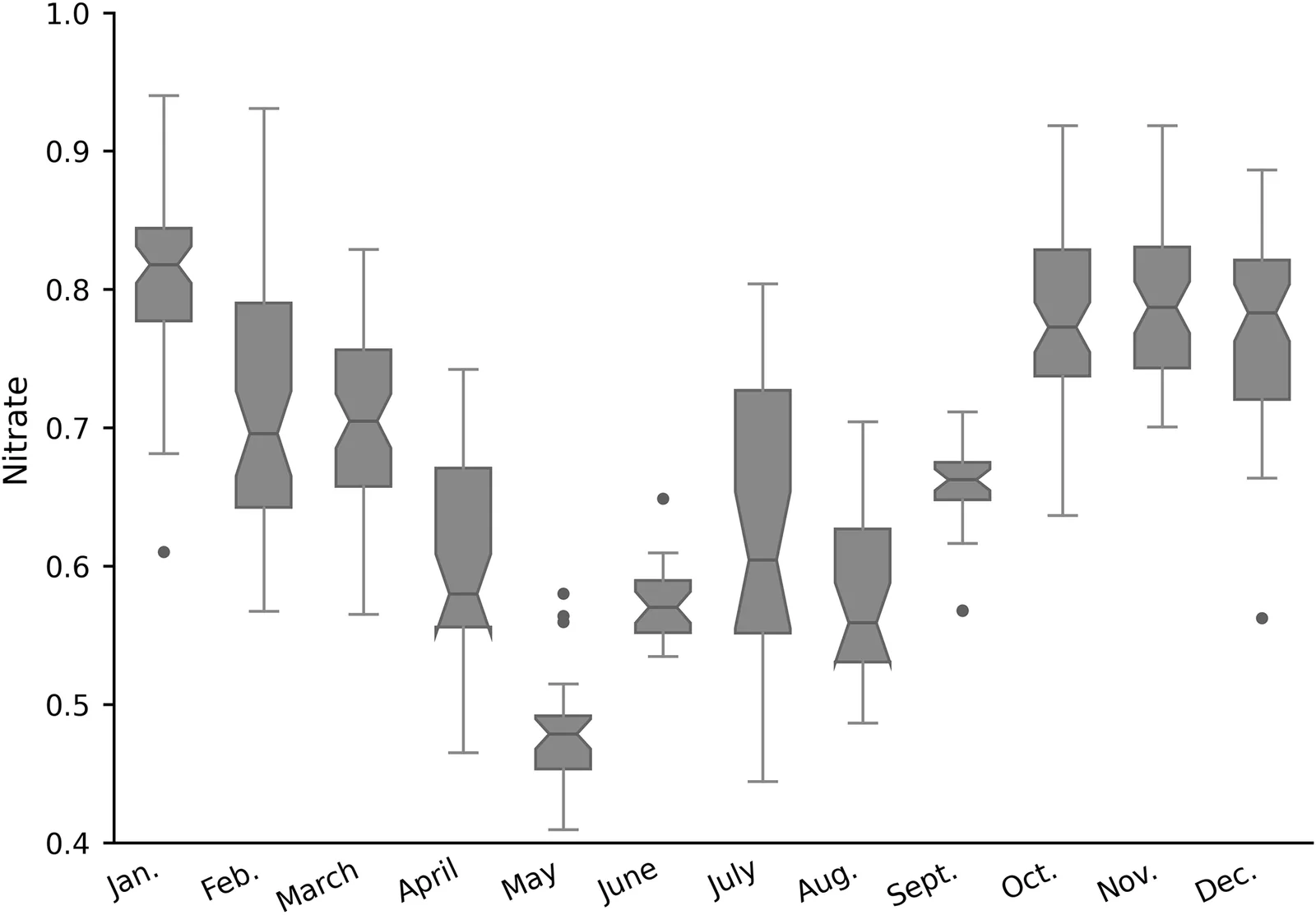
Nutrient concentrations at the Greenup Locks from a SUNA nitrate sensor deployed on the lock wall.

### Algal trends in the pool

Biological endpoints measured with the high frequency sensors or calculated using the high frequency dataset include chl-a, phyco, and the ratio of phyco to chl-a (BGG). These values are considered herein to represent green algae, cyanobacteria (blue-green algae), and the ratio of cyanobacteria to green algae. While no blooms occurred during the data collection period, an increasing ratio of cyanobacteria with respect to green algae is considered to represent conditions favoring cyanobacteria relative to other taxa and hence, conditions that may support HAB development. As noted previously, sensor measurements of fluorescence are converted to estimates of µg L^-1^ chlorophyll and phycocyanin concentrations are not consistently correlated with algal cell density or biomass [18]. Algal cells can regulate internal pigment concentrations based on intracellular and environmental conditions [19] which limits the applicability of the technology. Chlorophyll was generally low in readings from the lock wall with an average of 2.24 ug L^-1^ and a range from 0.56 to 18.47 ug L^-1^. Slightly higher monthly averages in April and August were the only seasonal trends noted although there were days with higher readings recorded throughout the year (Fig 9a). Cyanobacteria were highest in the winter and spring with the lowest averages in the summer and fall (Fig 9b). Phycocyanin ranged from 0.0 to 2.09 ug L^-1^ with an average of 0.38 ug L^-1^. The ratio of cyanobacteria to green algae demonstrated a similar seasonal trend as the cyanobacteria (Fig 9c) indicating that factors influencing cyanobacteria are driving the bloom potential. The ratio ranged from −0.27 to 0.87 with an average of 0.18.

**Fig 9:**
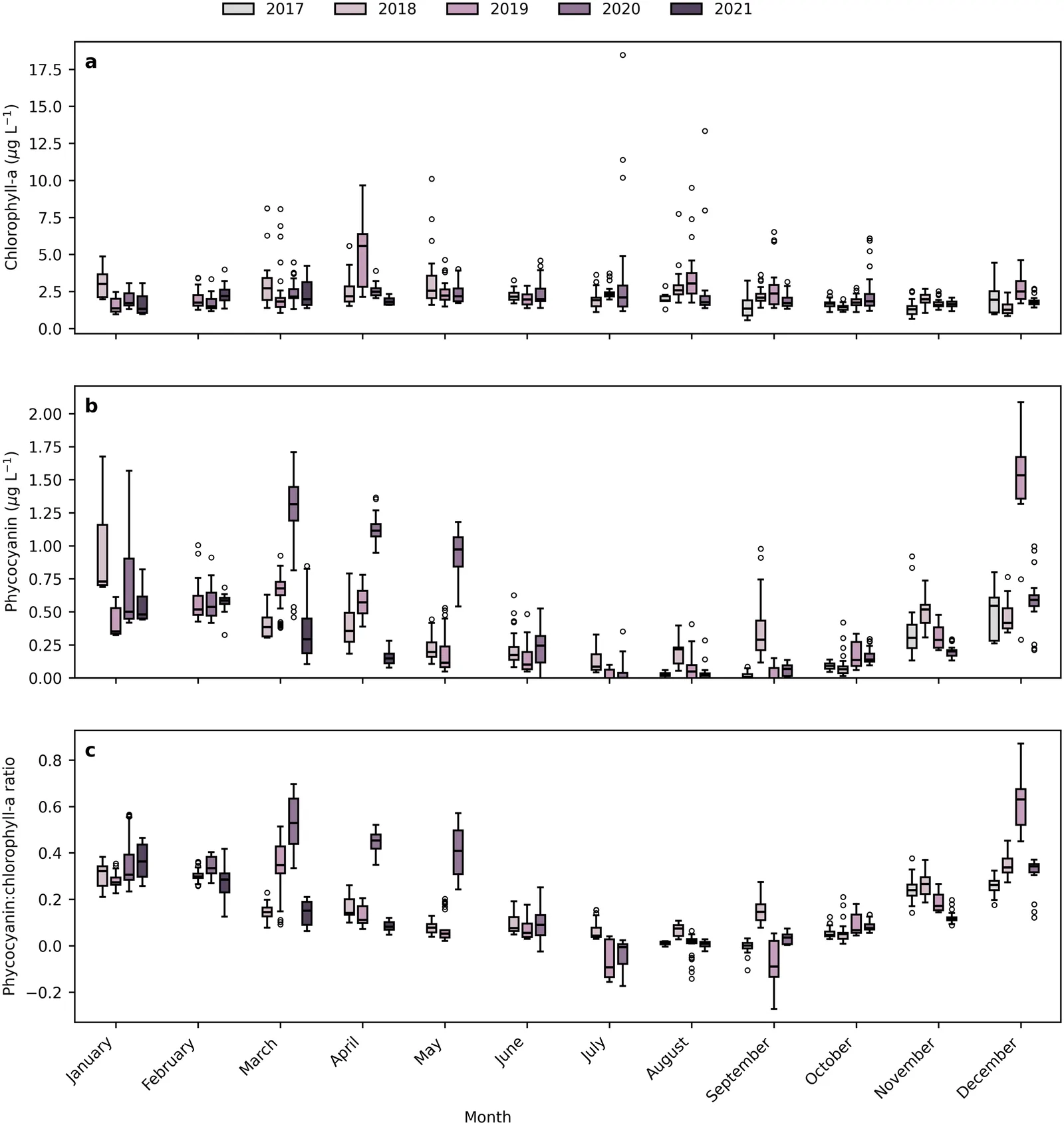
Seasonal trends in green algae (a) and cyanobacteria (b) occurrence, with cyanobacteria to green algae ratio (c), from 44 months of high frequency monitoring at Greenup Locks in the Ohio River.

Uniformity of temperature, turbidity, and other parameters described above for most of the pool was also shown in the distribution of chlorophyll and phycocyanin. Although generally low in concentration, chlorophyll was variable on different sampling dates and was generally uniform in distribution with increased fluorescence in the surface samples at the downstream end of the pool where light penetrated and turbidity was reduced (Fig 10). Cyanobacteria showed a similar uniform distribution and overall low concentrations with the exception of the August 2019 sampling event (Fig 10).

**Fig 10:**
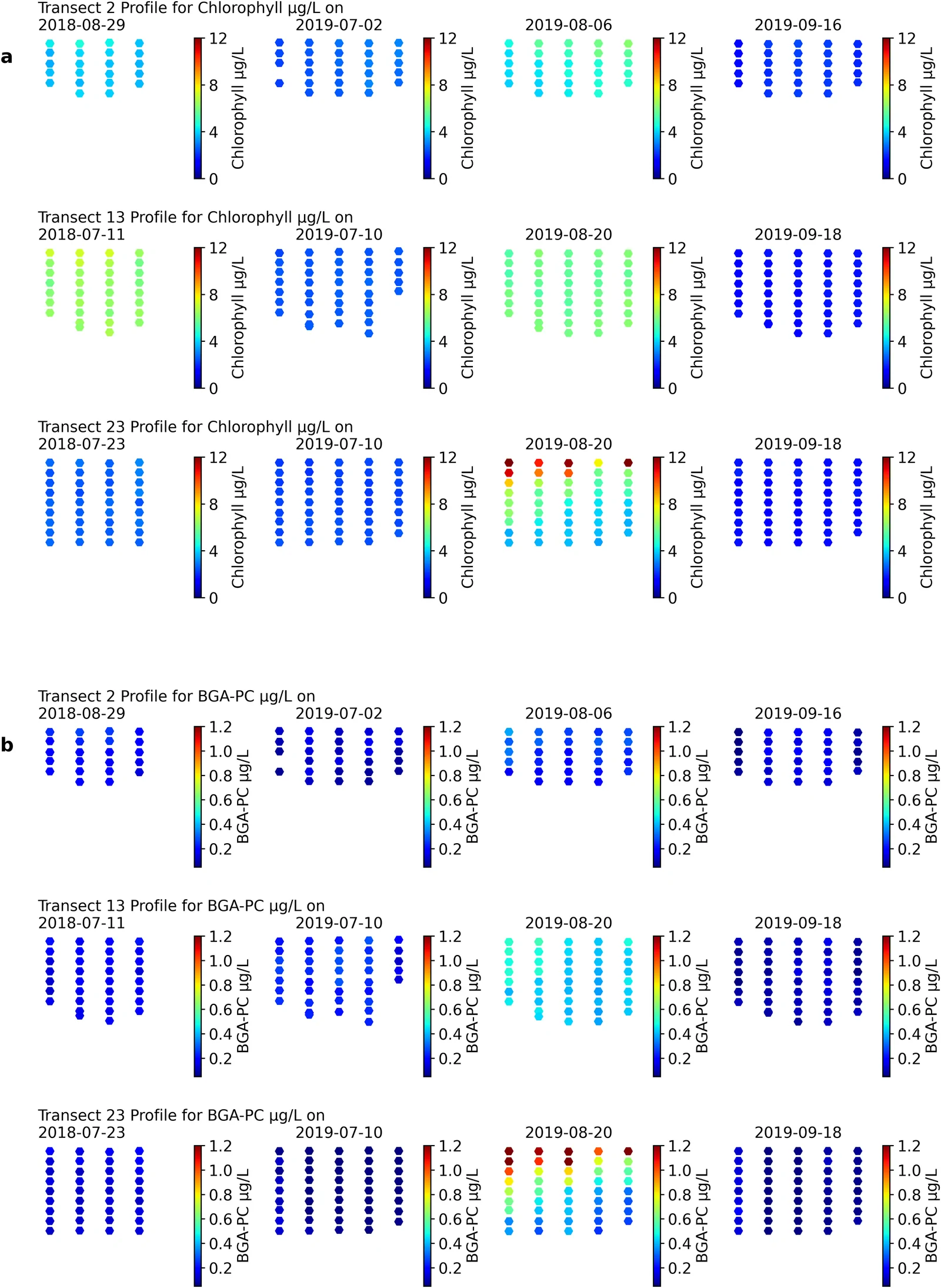
Spatial distribution of green algae (a) and cyanobacteria (b) at transects in the Greenup Pool of the Ohio River from sampling dates in the summer of 2018 and 2019.

A weak but significant correlation was found between chlorophyll and phycocyanin (ρ = 0.2767, p < 0.0001). However, there were a substantial number of samples with a higher chlorophyll concentration that did not have a corresponding increase in cyanobacteria (Fig 11). These values were associated with the highest green algae concentrations, generally between 5 and 20 ug L^-1^, and may indicate conditions favoring green algal growth relative to cyanobacteria. Correlation of available physical and chemical data with these highest chlorophyll values (Table 4), indicated significant relationships between chlorophyll and conductivity, pH, and DO % saturation supporting algal productivity in these samples. There was no relationship between these peak chlorophyll values and pool retention time or turbidity.

**Table 4:** Spearman correlation coefficients showing the relationship between physicochemical parameters and chlorophyll (green algae) from a subset of sampling dates with elevated green algae but not a corresponding elevation in cyanobacteria.

	r	df	p-value
Retention	−0.087	39	0.588
Turbidity	−0.049	39	0.761
PAR	0.390	32	0.023
Temperature	0.251	39	0.114
Conductivity	0.379	39	0.014
pH	0.465	39	0.002
DO saturation	0.455	39	0.003

**Fig 11:**
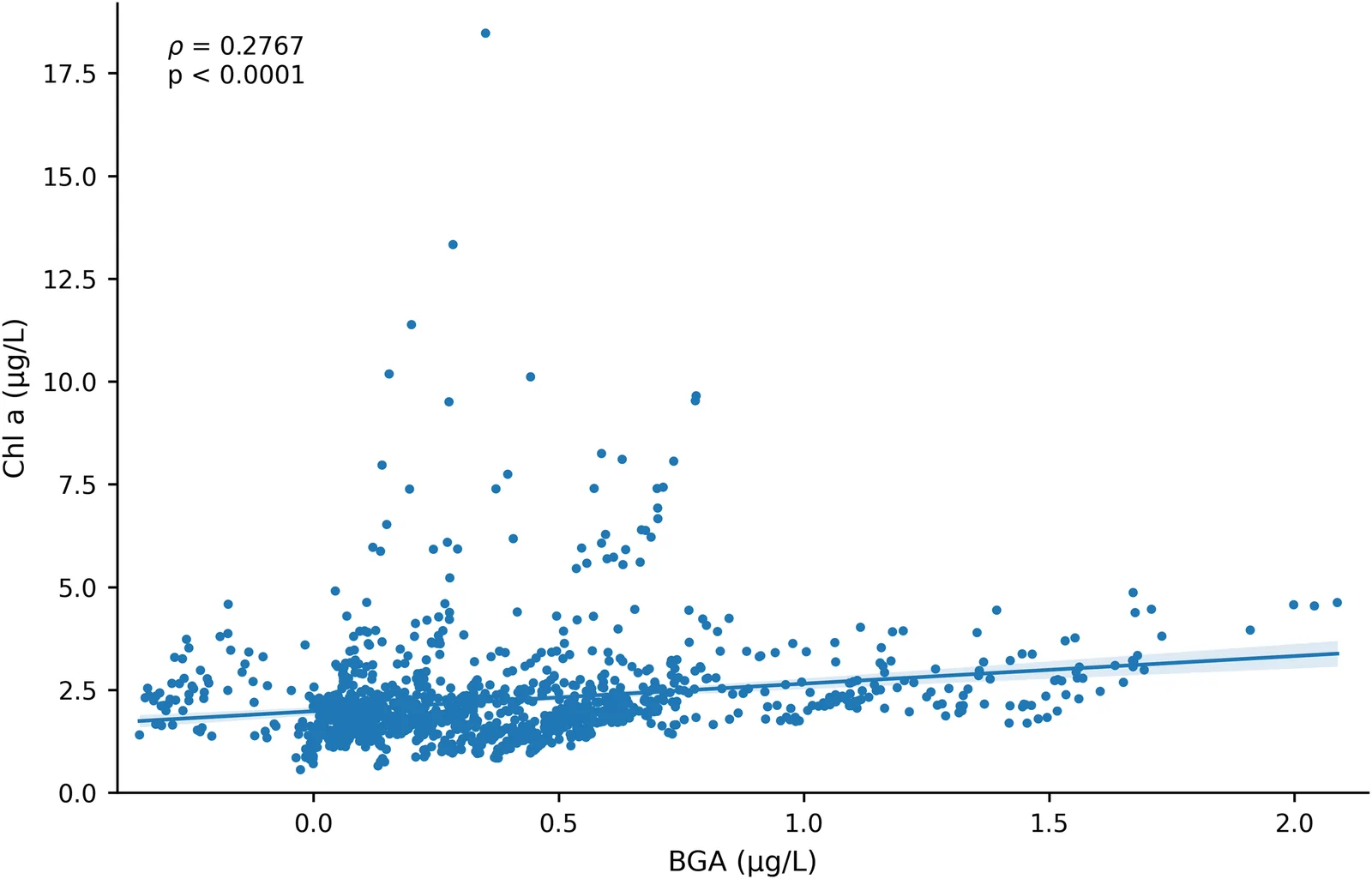
The relationship between chlorophyll and cyanobacteria is determined by Spearman Rank Correlation Coefficient. The correlation is weak but significant.

The daily dataset was used to evaluate the relationship between the concentration of chlorophyll, phycocyanin, and chlorophyll to phycocyanin ratio with physical and chemical parameters. Outliers, though identified, were not removed from the data set as they were deemed correct readings. Four of the five chl-a outliers that were identified by the Grubbs outlier test, the highest chl-a readings in the dataset, occurred from 7/29/20–8/1/20. The three highest phycocyanin measurements in the dataset were similarly identified as outliers by the Grubbs outlier test and occurred from 12/18/20–12/20/20.

In Spearman Correlation analysis of individual physical parameters, chl-a showed a weak but significant association with both pool retention time and temperature (Fig 12 and Table 5). Temperature and retention time were more strongly associated with the cyanobacteria and the BGG ratio. A similar trend was seen in turbidity where each of the three algal endpoints were significantly associated with the water quality indicator, again, the cyanobacteria more so than the green algae (Fig 12 and Table 5). PAR was significantly but weakly positively correlated with green algae but significantly negatively correlated with cyanobacteria, and the BGG ratio. This negative association could be due to changes in biomass, abundance or chromatic adaptation. It should also be noted that with the high frequency data set, significance levels are constrained by the large sample size and lack of independence of the variables. Interpretation of the high significance levels should proceed cautiously.

**Table 5:** Spearman correlations and significance values for physical parameters from the Greenup Pool of the Ohio River with algal endpoints.

	Retention			Temperature °C		
	r	df	p-value	r	df	p-value
Chl-a	−0.156	1171	<0.00001	0.132	1255	<0.00001
Phyco	−0.456	1171	<0.00001	−0.763	1255	<0.00001
BGGratio	−0.414	1134	<0.00001	−0.819	1217	<0.00001
	Turbidity			PAR		
	r	df	p-value	r	df	p-value
Chl-a	0.209	1255	<0.00001	0.296	560	<0.00001
Phyco	0.620	1255	<0.00001	−0.244	560	<0.00001
BGGratio	0.546	1217	<0.00001	−0.371	533	<0.00001

**Fig 12:**
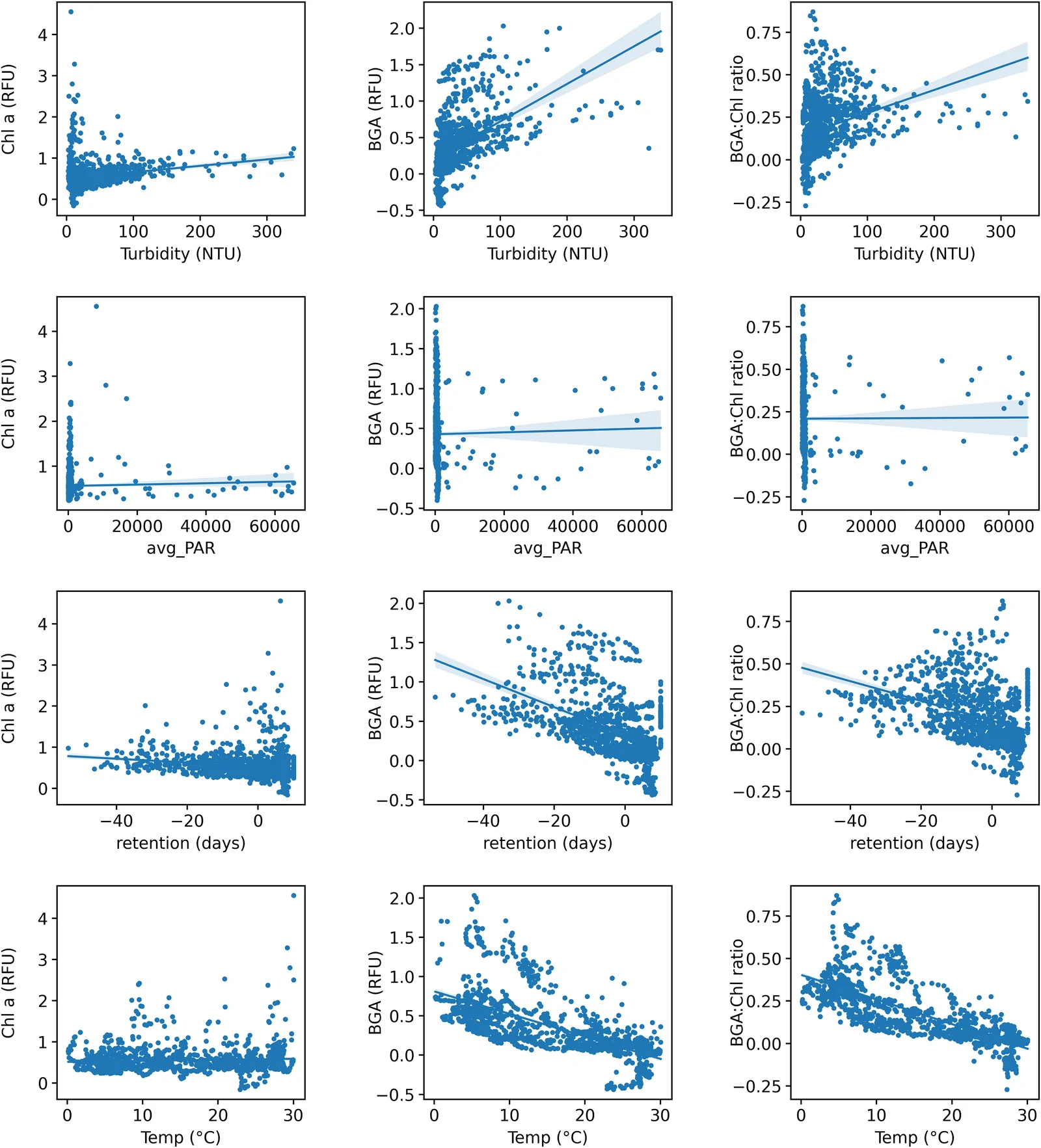
Relationship between green algae and cyanobacteria proxies, and the BGG ratio, with pool retention time, temperature, turbidity and PAR.

Spearman correlation analysis of individual chemical parameters demonstrated phycocyanin’s strong negative association with conductivity and strong positive correlations with oxygen (Table 6) which were different than chlorophyll relationships to these parameters. Green algae were not significantly correlated with conductivity, was only weakly associated with DO saturation, and was negatively associated with pH and dissolved oxygen concentration. There were no relationships between chl-a, phycocyanin or BGG ratio and either nitrate nitrogen or total phosphorus when compared with contemporary ORSANCO grab samples (Table 6).

**Table 6:** Spearman correlations and significance values for chemical parameters from the Greenup Pool of the Ohio River with algal endpoints.

	Conductivity			pH		
	r	df	p-value	r	df	p-value
Chl-a	−0.030	1255	0.284	−0.139	1255	<0.00001
Phyco	−0.534	1255	<0.00001	0.025	1255	0.368
BGGratio	−0.562	1255	<0.00001	0.042	1255	0.132
	DO mg L^-1^			DO % saturation		
	r	df	p-value	r	df	p-value
Chl-a	−0.069	1255	0.015	0.088	1255	0.00172
Phyco	0.749	1255	<0.00001	0.580	1255	<0.00001
BGGratio	0.787	1255	<0.00001	0.558	1255	<0.00001
	Nitrate-nitrogen			Phosphorus		
	r	df	p-value	r	df	p-value
Chl-a	−0.10	45	0.491	−0.10	45	0.513
Phyco	−0.15	45	0.308	0.189	45	0.204
BGGratio	−0.06	45	0.697	0.230	45	0.120

With highly variable results and multiple single parameter correlations, multiple regression analysis was used to further investigate data trends. Seasonal multiple regression models with serial correction were fit for chlorophyll, phycocyanin, and the cyanobacteria to chlorophyll ratio using days with complete data for temperature, turbidity, specific conductivity, pH, solar radiation, nitrate, and pool retention time. Sample size ranged from 88 to 135 in the trimmed seasonal datasets. Dissolved oxygen saturation, which is responsive to algal productivity but is not a driver of growth, was not included in the analysis. Results are presented separately for phycocyanin, chlorophyll, and cyanobacteria to chlorophyll ratio in Tables 7–9. Phycocyanin concentrations were significantly associated with physicochemical variables in all seasonal multiple regression models (Table 7). Model strength was highest in spring (R^2^ = 0.8769, F = 987.41, p < 0.0001), followed by fall (R^2^ = 0.6985, F = 33.103, p < 0.001), winter (R^2^ = 0.5583, F = 22.568, p < 0.001), and summer (R^2^ = 0.2057, F = 2.922, p = 0.0090). Turbidity was positively associated with phycocyanin in spring, fall, and winter, while temperature was negatively associated with phycocyanin in spring and fall. In summer, pH was the only significant predictor and was positively associated with phycocyanin. In winter, pH was negatively associated with phycocyanin, while turbidity was positively associated. Retention time, nitrate, specific conductance, and solar radiation were not consistently significant across seasons (Fig 13).

**Table 7:** Annual and seasonal multiple regression analysis of physicochemical variables with phycocyanin concentrations.

Model subset	R²	F	Model p-value	Predictor	t value	Predictor p-value
Spring	0.8769	98.741	<0.0001	pH	0.101	0.9201
				Temperature	−3890	0.0002
				Specific conductance	−0.107	0.9152
				Retention time	0.203	0.8395
				Nitrate	−1.358	0.1775
				Solar radiation	−0.514	0.6082
				Turbidity	20.656	<0.0001
Summer	0.2057	2.922	0.0090	Solar radiation	−1.160	0.2494
				Retention time	−0.340	0.7349
				Turbidity	1.168	0.2464
				Temperature	−0.146	0.5846
				pH	4.164	0.0001
				Specific conductance	−1.180	0.2414
				Nitrate	1.451	0.1507
Fall	0.6985	33.103	<0.0001	Retention time	−1.890	0.0617
				Temperature	−8.074	<0.0001
				Nitrate	1.158	0.2496
				Specific conductance	1.062	0.2909
				pH	−0.442	0.6597
				Solar radiation	−0.170	0.8654
				Turbidity	2.436	0.0166
Winter	0.5583	22.568	<0.0001	pH	−4.628	<0.0001
				Temperature	0.754	0.4539
				Specific conductance	0.101	0.9195
				Retention time	−1.473	0.1433
				Nitrate	1.340	0.1828
				Turbidity	4.579	<0.0001
				Solar radiation	0.323	0.7470

**Table 8:** Annual and seasonal multiple regression analysis of physicochemical variables with chlorophyll concentrations.

Model subset	R²	F	Model p-value	Predictor	t value	Predictor p-value
Spring	0.5715	18.484	<0.0001	Specific conductance	2.509	0.0138
				Turbidity	10.734	<0.0001
				Temperature	0.981	0.3288
				Nitrate	−0.542	0.5890
				Retention time	1.580	0.1175
				pH	0.123	0.9024
				Solar radiation	−1.044	0.2990
Summer	0.4259	8.371	<0.0001	pH	6.172	<0.0001
				Retention time	−0.691	0.4917
				Temperature	2.274	0.0257
				Nitrate	0.079	0.9370
				Solar radiation	−2.138	0.0356
				Turbidity	2.217	0.0295
				Specific conductance	−1.215	0.2279
Fall	0.1664	2.851	0.0094	Retention time	−2.188	0.0310
				Solar radiation	−1.270	0.2069
				Temperature	−0.473	0.6369
				Turbidity	1.796	0.0754
				Specific conductance	0.0039	0.9693
				pH	−0.550	0.5835
				Nitrate	−0.397	0.6919
Winter	0.6908	39.891	<0.0001	Turbidity	13.324	<0.0001
				Nitrate	1.893	0.0607
				Specific conductance	1.834	0.0690
				pH	0.344	0.7314
				Temperature	−0.552	0.5819
				Solar radiation	−2.274	0.0247
				Retention time	−2.436	0.0162

**Table 9:** Annual and seasonal multiple regression analysis of physicochemical variables with the phycocyanin:chlorophyll ratio.

Model subset	R²	F	Model p-value	Predictor	t value	Predictor p-value
Spring	0.3160	6.402	<0.0001	pH	−1.520	0.1318
				Temperature	−4.607	<0.0001
				Turbidity	−2.915	0.0044
				Retention time	0.124	0.9012
				Solar radiation	2.384	0.0191
				Specific conductance	−1.178	0.2415
				Nitrate	−1.605	0.1117
Summer	0.0849	1.048	0.4052	Solar radiation	0.432	0.6668
				Temperature	−1.451	0.1508
				Turbidity	0.344	0.7318
				pH	1.122	0.2657
				Retention time	−0.586	0.5598
				Nitrate	1.5530	0.1244
				Specific conductance	0.490	0.6256
Fall	0.5804	19.761	<0.0001	Temperature	−8.212	<0.0001
				Retention time	−0.853	0.3957
				Specific conductance	2.043	0.0436
				Nitrate	−1.401	0.1643
				pH	0.201	0.8412
				Turbidity	−0.610	0.5430
				Solar radiation	0.642	0.5221
Winter	0.3544	9.804	<0.0001	pH	−4.981	<0.0001
				Temperature	0.934	0.3521
				Retention time	2.753	0.0068
				Turbidity	−3.695	0.0003
				Specific conductance	−0.680	0.4979
				Solar radiation	2.995	0.0033
				Nitrate	−0.041	0.9667

**Fig 13:**
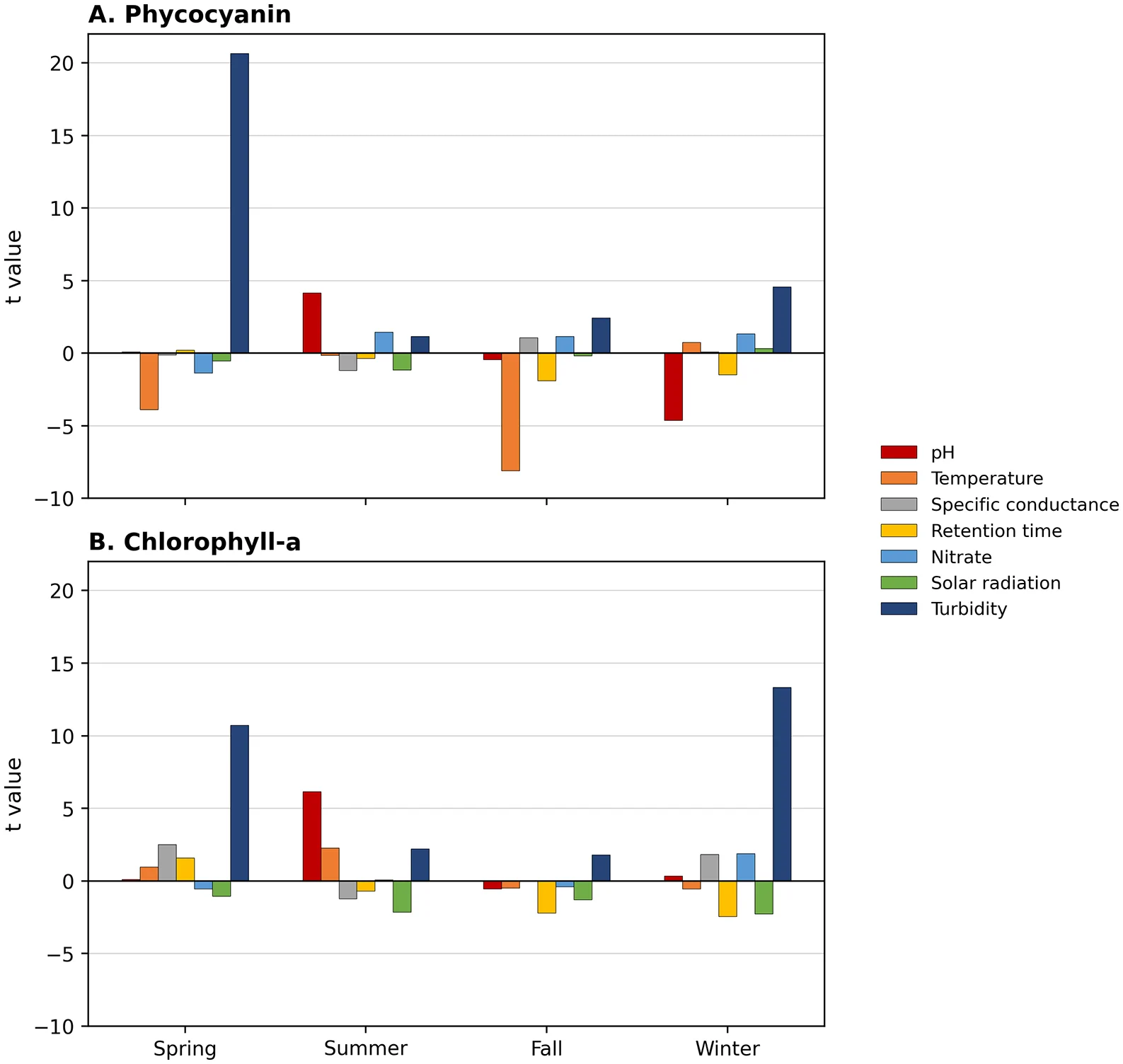
Relative contribution and seasonal variability of environmental drivers on phycocyanin (a) and chlorophyll (b) concentrations indicated by t-values of seasonal multiple regression analysis. For signiﬁcance values, see Tables 7 and 8.

Chlorophyll concentrations were significantly associated with physicochemical variables in all seasonal multiple regression models (Table 8). Model strength was highest in winter (R^2^ = 0.6908, F = 39.891, p < 0.0001), followed by spring (R^2^ = 0.5717, F = 18.484, p < 0.001), summer (R^2^ = 0.4259, F = 8.371, p < 0.0001), and fall (R^2^ = 0.1664, F = 2.851, p = 0.0094). Turbidity was positively associated with chlorophyll in spring, summer, and winter. In spring, specific conductance was also positively associated with chlorophyll. In summer, chlorophyll was positively associated with pH, temperature, and turbidity, and negatively associated with solar radiation. In fall, retention time was the only significant predictor and was negatively associated with chlorophyll. In winter, chlorophyll was positively associated with turbidity and negatively associated with solar radiation and retention time. Nitrate was not a significant predictor in any seasonal chlorophyll model (Fig 13).

The phycocyanin:chlorophyll ratio was significantly associated with physicochemical variables in spring, fall, and winter, but not summer (Table 9). Model strength was highest in fall (R^2^ = 0.5804, F = 19.761, p < 0.0001), followed by winter (R^2^ = 0.3544, F = 9.804, p < 0.0001), and spring (R^2^ = 0.3160, F = 6.402, p < 0.0001). In spring, the ratio was negatively associated with temperature and turbidity and positively associated with solar radiation. In fall, the ratio was negatively associated with temperature and positively associated with specific conductance. In winter, the ratio was negatively associated with pH and turbidity and positively associated with retention time and solar radiation. No individual predictors were significant in the summer ration model.

## Discussion

Our study reports a 44-month high frequency data set with physical, chemical and biological transect sampling to provide an in-depth evaluation of a run of river pool in a managed river ecosystem. The large dataset evaluated with multiple analytical techniques reveals important trends in the distribution of green algae and cyanobacteria within the Greenup Pool. In addition to describing seasonal physical and chemical conditions, relationships between chemical and physical endpoints with biological endpoints informs on variables significantly influencing algal community dynamics.

Factors correlated with both phycocyanin, and chlorophyll were identified with seasonal variability in the importance of physical and chemical variables to each type of algal indicator. Having experienced no blooms during the study period, the ratio of phycocyanin to chlorophyll is included indicating environmental conditions favoring cyanobacteria productivity over green algae/diatoms that may lead to bloom development. Consistently, the ratio was driven by increases in the cyanobacteria concentration with green algae and diatoms having little effect on the ratio. This lack of influence of the denominator may indicate that competition with the green algae and diatoms was not significant under the conditions represented. Parameters associated with the cyanobacteria growth vary seasonally but were consistently significantly inversely related to temperature in the spring and fall seasons Multiple parameters were influential on the model in the winter when cyanobacteria peaked. While predictive ability was demonstrated in all seasons, summer predictions better represented green algae than cyanobacteria and pH, temperature, solar radiation and turbidity all contributing to the model. Although turbidity is expected to impair algal growth by reducing light penetration, the relationship between cyanobacteria growth and pH is more complicated [20]. Elevated cyanobacteria abundance may result in increased pH [21] and thus, pH may be useful in HAB detection and prediction. Additionally, higher temperatures are thought to favor cyanobacteria [22] as they are tolerant of higher temperatures. Our evaluation supports this but also provides evidence of cooler temperature maximums for the cyanobacteria as indicated by previous researchers [23].

Foundational knowledge of spatial and temporal variability in the ecosystem is necessary to inform efficient predictions, forecasting and management of riverine HABs and designing physical monitoring and remote sensing strategies for the pool. These findings contribute to the body of knowledge on algal communities in run of river impoundments. They also inform monitoring strategy development, support parameterization of models and predictive tools, and identify appropriate endpoints for inclusion in modeling, monitoring and management.

With increasing frequency and severity of HABs in riverine ecosystems, research has focused on the conditions supporting blooms in these systems [18,24–27] and on the contribution of rivers to bloom formation in lakes [28] and coastal ecosystems [29]. The propensity of rivers to support blooms is influenced by multiple factors including the variables well described from lake ecosystems; light, temperature, and nutrients, but with additional influence from the downstream transport of plankton [18,25] and the turbidity associated with large river systems [30]. Further, few large rivers are unmanaged or freely flowing such that there are multiple management strategies for consideration.

Many studies focus on HAB monitoring, detecting, forecasting and predicting using high frequency sensors such as the EXO sonde used in our data collection [31]. Generally, these studies report chlorophyll as proxy for algal abundance or biomass [31,32]. One noteworthy finding of this analysis is the difference between the response of phytoplankton represented by chlorophyll and phycocyanin. While the sensors may detect both, the low levels of phycocyanin measured have caused previous studies to favor chlorophyll over phycocyanin as an algal indicator. However, our results concur with studies that indicate additional processing is necessary to enable that assumption [18,24,33]. Phycocyanin sensors have been found to have slightly lower reliability as compared with the chlorophyll sensor. Wong and Hobbs reported a 75% data reliability finding for the phycocyanin sensor as compared to the 95% reliability finding for the chlorophyll sensor in field collected data [34]. In comparisons with laboratory analysis, they reported phycocyanin fluorescence was strongly correlated with laboratory phycocyanin (r^2^ = 0.78), chlorophyll (r^2^ = 0.89), and microcystin concentrations (r^2^ = 0.85) while the sonde chlorophyll concentration was not significantly correlated with the laboratory chlorophyll concentration or the cyanobacteria indicators of phycocyanin and microcystin. Similar results are reported by Rome and others with a correlation of coefficient of 0.81 relating total cyanobacteria cells per milliliter of water and phycocyanin relative fluorescence units [35]. Some studies have successfully utilized phycocyanin sensors for predicting cell density and microcystin [36] while other studies were successful only at relating phycocyanin fluorescence with biovolume and not cyanobacteria cell density [37]. Despite the limitations of the phycocyanin sensors, the chlorophyll sensor does not reliably fluoresce in response to phycocyanin [35,38,39] and thus use of the chlorophyll measurement may not detect or adequately predict environmental parameters driving cyanobacteria HABs. We report similar findings here. While our analysis shows correlations, not causation, variables associated with increases in phycocyanin, specific to cyanobacteria, are not necessarily those associated with chlorophyll concentrations in our dataset. Thus, focusing on chlorophyll for HABs monitoring and management strategies may not be sufficient for early detection and predicting conditions supporting cyanobacteria growth and blooms.

Several parameters evaluated demonstrated differences between chlorophyll and phycocyanin that may give insight into conditions favoring cyanobacteria, and potentially HAB formation, as compared to the green algae and diatoms also represented in chlorophyll measurements. The most substantial of these discrepancies is the relationship of algal proxies to temperature. It is widely reported that cyanobacteria are favored under warmer conditions [18,22], and that may be true for some taxa and some temperatures, but our study found the highest phycocyanin concentrations in the winter and spring. This was also a period of seasonally high turbidity and flow. While this increase may have indicated adaptation to the higher turbidity, there is potential for cyanobacteria growth and bloom development under cooler temperatures [23]. Further, community development under the colder conditions may may influence availability of phytoplankton and cyanobacteria dynamics later in the warmer seasons. The role of cold water algal growth on warmwater blooms should be further investigated.

The profile-derived light attenuation values reported here are consistent with prior Ohio River light-climate estimates. Bukaveckas et al. [40] reported Ohio River attenuation coefficients of 1.75 ± 0.07 m^−1^, with direct measurements from the McAlpine and Smithland Pools averaging 1.77 ± 0.27 m^−1^. In the present study, mean kd was 1.829 m^−1^, and the mean 1% light depth was 3.188 m, closely matching the previously reported Ohio River 1% light depth of approximately 3.2 m [40]. This agreement indicates that the Greenup Pool light environment was broadly consistent with prior mainstem Ohio River observations, while also showing substantial profile-level variability in light attenuation.

The Ohio River is known to be light limited [41] with light levels impaired by turbidity of at least 5 NTU [42] 95% of the time. As Koch and others suggested, nutrient limitations may only drive algal communities when light is not limiting [41]. We also found in the Greenup Pool of the Ohio River, that nutrient levels sufficient to support algal growth were generally available throughout the sampling period. As indicated previously, our nitrate monitoring showed an average value of 0.710 mg L^-1^ and a minimum value of 0.410 mg L^-1^. This is similar to ORSANCO’s bimonthly nutrient database that reported an average of 0.770 mg L^-1^ and a minimum nitrate concentration of 0.420 mg L^-1^ over a 10-year sampling period. ORSANCO also reports a minimum phosphorus concentration of 0.010 mg L^-1^ and an average of 0.086 mg L^-1^. The lowest phosphorus recorded is consistent with the reported cyanobacteria growth threshold of 0.010 mg L^-1^ [43] suggesting phosphorus limitation was not prevalent. Although nutrient limitation was not observed, it may still be a factor in algal community dynamics. For example, nutrients may become limited under critical low flow, high light conditions when algal growth increases. Further evaluation of the ORSANCO database indicated significant Spearman rank order correlations between total phosphorus and both total nitrogen (ρ = 0.56, n = 104, p < 0.0001) and dissolved inorganic nitrogen (ρ = 0.28, n = 120, p = 0.0023) which are important for developing predictive scenarios. Total nitrogen and phosphorus were also correlated with total suspended solids and flow indicating seasonal variability and potentially indicating nonpoint sources.

Another significant finding of our study was the demonstration of spatial and temporal variability of the conditions within the single pool. Transect sampling, which occurred during the lowest flow summer conditions, demonstrates the prevalence of turbidity in the mainstem of the Ohio River and we report evidence of limited stratification and settling of turbidity occurring concurrently with higher light penetration. Transect data evaluation generally indicates variability between but not within transects indicating mixing and isothermal conditions throughout most of the sampling period and locations. However, in addition to the depth increasing in a downstream direction, limited water column stratification was demonstrated at the downstream transects in August 2019. Defined by Davis and others as a temperature differential greater than 0.5^o^C [17], temperature stratification occurred on multiple sampling dates at the downstream transects. Higher chlorophyll readings at surface locations when suspended sediment settling was also noted supports the significance of turbidity limiting algal growth. The potential for water column stratification indicates variability in velocity and water residence time that may be influential in algal communities and may offer early warning opportunities for bloom conditions or management levers which require further investigation.

Characterization of the Greenup Pool of the Ohio River describes physical, chemical and biological conditions in a managed river ecosystem and relates parameters to biological endpoints. With a focus on HAB potential, the variables likely important in algal growth and bloom development are primarily light and temperature with turbidity substantially influencing light availability. The pool is found to be well mixed and uniform under most conditions with very limited spatial and temporal opportunity for stratification that may influence algal community structure and support productivity. However, low flow in the river may provide opportunities for algal growth. Definition of these conditions may inform monitoring and management of HABs in the regulated river system. Further, clarification of environmental conditions favoring cyanobacteria over green algae and diatoms, and the importance of monitoring and modeling cyanobacteria specific endpoints for detection and management are demonstrated.

## Supporting information

S1 Table Variance Inflation Factors from collinearity analysis of predictor variables used in multiple regression models. (XLSX)

S2 Table Correlation coefficients for examining collinearity of predictor variables used in seasonal multiple regression analysis. (XLSX)

S3 Table Autocorrelation analysis before and after multiple regression models with AR(1) serial-correlation correction. (XLSX)

S4 Table Breusch-Pagan test for heteroscedasticity results from the residuals of the seasonal multiple regression with serial correction. (XLSX)

## References

[1] Vannote RL, Minshall GW, Cummins KW, Sedell JR, Cushing CE. The river continuum concept. Can J Fish Aquat Sci. 1980;37(1):130–7. doi: 10.1139/f80-017

[2] Wehr JD, Thorp JH. Effects of navigation dams, tributaries, and littoral zones on phytoplankton communities in the Ohio River. Can J Fish Aquat Sci. 1997;54(2):378–95. doi: 10.1139/f96-283

[3] U.S. Environmental Protection Agency. The effects: Dead zones and harmful algal blooms [Internet]; 2023 [cited 2023 Oct 28]. Available from: https://www.epa.gov/nutrientpollution/effects-dead-zones-and-harmful-algal-blooms

[4] Weigelhofer G, Hein T, Bondar-Kunze E. Phosphorus and nitrogen dynamics in riverine systems: Human impacts and management options. In: Schmutz S, Sendzimir J, editors. Riverine ecosystem management. Cham: Springer; 2018. p. 187–202. doi: 10.1007/978-3-319-73250-3_10

[5] Wehr JD, Descy JP. Use of phytoplankton in large river management. J Phycol. 1998;34(5):741–9. doi: 10.1046/j.1529-8817.1998.340741.x

[6] O’Neill KM. Rivers by design: State power and the origins of US flood control. Durham: Duke University Press; 2006.

[7] Dokulil MT. Environmental control of phytoplankton productivity in turbulent turbid systems. Hydrobiologia. 1994;289(1–3):65–72. doi: 10.1007/bf00007409

[8] Kennedy JT, Whalen SC. Seasonality and controls of phytoplankton productivity in the middle Cape Fear River, USA. Hydrobiologia. 2007;598(1):203–17. doi: 10.1007/s10750-007-9151-9

[9] Ohio River Valley Water Sanitation Commission. Biennial Assessment of Ohio River Water Quality Conditions 2014-2018 [Internet]; 2020 [cited 2023 Oct 28]. Available from: https://www.orsanco.org/wp-content/uploads/2020/06/ORSANCO_2020_305b_Report.pdf

[10] U.S. Environmental Protection Agency. Recommended human health recreational ambient water quality criteria or swimming advisories for microcystins and cylindrospermopsin [Internet]. Washington (DC): U.S. Environmental Protection Agency; 2019 [cited 2026 Jul 16]. Available from: https://www.epa.gov/wqc/recreational-water-quality-criteria-and-methods

[11] Youngstrom G. ORSANCO Harmful Algae Bloom Monitoring, Response and Communication Plan [Internet]; 2021 [cited 2023 Oct 28]. Available from: https://www.orsanco.org/wp-content/uploads/2021/02/FINAL-2021-HAB-Monitoring-and-Response-Plan.pdf

[12] Hardy B. Harmful algal blooms in the Ohio River: Spatial and temporal differences. Louisville (KY): University of Louisville; 2021.

[13] O’Brien RM. A caution regarding rules of thumb for variance inflation factors. Qual Quant. 2007;41:673–90. doi: 10.1007/s11135-006-9018-6

[14] Dormann CF, Elith J, Bacher S, Buchmann C, Carl G, Carré G, et al. Collinearity: A review of methods to deal with it and a simulation study evaluating their performance. Ecography. 2012;36(1):27–46. doi: 10.1111/j.1600-0587.2012.07348.x

[15] Schreiber SG, Schreiber S, Tanna RN, Roberts DR, Arciszewski TJ. Statistical tools for water quality assessment and monitoring in river ecosystems – A scoping review and recommendations for data analysis. Water Qual Res J. 2022;57(1):40–57. doi: 10.2166/wqrj.2022.028

[16] Seabold S, Perktold J. Statsmodels: Econometric and statistical modeling with Python. Proceedings of the 9th Python in Science Conference; 2010. p. 92–6. 10.25080/Majora-92bf1922-011

[17] Davis DC, Facey JA, Brooks AJ, Westhorpe DP, Balzer M, Williamson N, et al. Critical flow velocity thresholds for preventing persistent thermal stratification and cyanobacterial blooms in rivers. Harmful Algae. 2025;149:102962. doi: 10.1016/j.hal.2025.102962 40935522

[18] Johnston BD, Finkelstein KM, Gifford SR, Stouder MD, Nystrom EA, Savoy PR, et al. Evaluation of sensors for continuous monitoring of harmful algal blooms in the Finger Lakes region, New York, 2019 and 2020. Reston (VA): U.S. Geological Survey; 2024. Scientific Investigations Report 2024-5010. doi: 10.3133/sir20245010

[19] YSI Incorporated. EXO user manual. Yellow Springs (OH): YSI Incorporated. [cited 2026 Jul 16]. Available from: https://www.ysi.com/file%20library/documents/manuals/exo-user-manual-web.pdf

[20] Glibert PM. Harmful algae at the complex nexus of eutrophication and climate change. Harmful Algae. 2020;91:101583. doi: 10.1016/j.hal.2019.03.001 32057336

[21] Huisman J, Codd GA, Paerl HW, Ibelings BW, Verspagen JMH, Visser PM. Cyanobacterial blooms. Nat Rev Microbiol. 2018;16(8):471–83. doi: 10.1038/s41579-018-0040-1 29946124

[22] Paerl HW, Huisman J. Blooms like it hot. Science. 2008;320(5872):57–8. doi: 10.1126/science.1155398 18388279

[23] Reinl KL, Harris TD, North RL, Almela P, Berger SA, Bizic M, et al. Blooms also like it cold. Limnol Oceanogr Lett. 2023;8(4):546–64. doi: 10.1002/lol2.10316

[24] Schmaedel NM, Harvey JW, Choi J, Stackpoole SM, Graham JL, Murphy JC. River control points for algal productivity revealed by transport analysis. Geophys Res Lett. 2024;51(5):e2023GL105137. doi: 10.1029/2023GL105137

[25] Bruns NE, Heffernan JB, Ross MRV, Doyle M. A simple metric for predicting the timing of river phytoplankton blooms. Ecosphere. 2022;13(12):e4348. doi: 10.1002/ecs2.4348

[26] Giblin SM, Gerrish GA. Environmental factors controlling phytoplankton dynamics in a large floodplain river with emphasis on cyanobacteria. River Res Appl. 2020;36(7):1137–50. doi: 10.1002/rra.3658

[27] Manier JT, Haro RJ, Houser JN, Strauss EA. Spatial and temporal dynamics of phytoplankton assemblages in the upper Mississippi River. River Res Appl. 2021;37(10):1451–62. doi: 10.1002/rra.3852

[28] Bridgeman TB, Chaffin JD, Filbrun JE. A novel method for tracking western Lake Erie Microcystis blooms, 2002-2011. J Great Lakes Res. 2013;39:83–9. doi: 10.1016/j.jglr.2012.11.004

[29] Jassby AD, Cloern JE, Cole BE. Annual primary production: Patterns and mechanisms of change in a nutrient-rich tidal ecosystem. Limnol Oceanogr. 2002;47(3):698–712. doi: 10.4319/lo.2002.47.3.0698

[30] Sellers T, Bukaveckas PA. Phytoplankton production in a large, regulated river: A modeling and mass balance assessment. Limnol Oceanogr. 2003;48(4):1476–87. doi: 10.4319/lo.2003.48.4.1476

[31] Boyer JN, Kelble CR, Ortner PB, Rudnick DT. Phytoplankton bloom status: Chlorophyll a biomass as an indicator of water quality condition in the southern estuaries of Florida, USA. Ecol Indic. 2009;9(6):S56–67. doi: 10.1016/j.ecolind.2008.11.013

[32] Jankowski KJ, Houser JN, Scheuerell MD, Smits AP. Warmer winters increase the biomass of phytoplankton in a large floodplain river. J Geophys Res Biogeosci. 2021. doi: 10.1029/2020JG006135

[33] Foster GM, Graham JL, King LR. Spatial and temporal variability of harmful algal blooms in Milford Lake, Kansas, May through November 2016. Reston (VA): U.S. Geological Survey; 2019. Scientific Investigations Report 2018-5166. doi: 10.3133/sir20185166

[34] Wong S, Hobbs WO. Exploring the use of fluorometric sensors to monitor harmful algal blooms in lakes [Internet]. Olympia (WA): Washington State Department of Ecology; 2019. Publication 20-03-010. Available from: https://fortress.wa.gov/ecy/publications/SummaryPages/2003010.html

[35] Rome M, Beighley RE, Faber T. Sensor-based detection of algal blooms for public health advisories and long-term monitoring. Sci Total Environ. 2021;767:144984. doi: 10.1016/j.scitotenv.2021.144984 33636761 PMC9562998

[36] Caraballo YA, Wu M. Summer phytoplankton assemblages and harmful cyanobacterial blooms in freshwaters of New Jersey. Northeast Nat. 2023;30(3):269–94. doi: 10.1656/045.030.0301

[37] Bowling LC, Zamyadi A, Henderson RK. Assessment of in situ fluorometry to measure cyanobacterial presence in water bodies with diverse cyanobacterial populations. Water Res. 2016;105:22–33. doi: 10.1016/j.watres.2016.08.051 27592302

[38] Seppälä J, Ylöstalo P, Kaitala S, Hällfors S, Raateoja M, Maunula P. Ship-of-opportunity based phycocyanin fluorescence monitoring of the filamentous cyanobacteria bloom dynamics in the Baltic Sea. Estuar Coast Shelf Sci. 2007;73(3–4):489–500. doi: 10.1016/j.ecss.2007.02.015

[39] Zamyadi A, Choo F, Newcombe G, Stuetz R, Henderson RK. A review of monitoring technologies for real-time management of cyanobacteria: Recent advances and future direction. TrAC Trends Anal Chem. 2016;85:83–96. doi: 10.1016/j.trac.2016.06.023

[40] Bukaveckas PA, MacDonald A, Aufdenkampe A, Chick JH, Havel JE, Schultz R, et al. Phytoplankton abundance and contributions to suspended particulate matter in the Ohio, Upper Mississippi and Missouri Rivers. Aquat Sci. 2011;73(3):419–36. doi: 10.1007/s00027-011-0190-y

[41] Koch RW, Guelda DL, Bukaveckas PA. Phytoplankton growth in the Ohio, Cumberland and Tennessee Rivers, USA: Inter-site differences in light and nutrient limitation. Aquat Ecol. 2004;38:17–26. doi: 10.1023/B:AECO.0000021082.42784.03

[42] Lloyd DS, Koenings JP, Laperriere JD. Effects of turbidity in fresh waters of Alaska. N Am J Fish Manag. 1987;7(1):18–33. doi: 10.1577/1548-8659(1987)7<18:EOTIFW>2.0.CO;2

[43] Steinberg CEW, Hartmann HM. Planktonic bloom-forming cyanobacteria and the eutrophication of lakes and rivers. Freshw Biol. 1988;20(2):279–87. doi: 10.1111/j.1365-2427.1988.tb00452.x

